# *Acinetobacter baumannii* represses type VI secretion system through a manganese-dependent small RNA-mediated regulation

**DOI:** 10.1128/mbio.03025-24

**Published:** 2024-12-20

**Authors:** Somok Bhowmik, Avik Pathak, Shivam Pandey, Kuldip Devnath, Abhiroop Sett, Nishant Jyoti, Timsy Bhando, Jawed Akhter, Saurabh Chugh, Ramandeep Singh, Tarun Kumar Sharma, Ranjana Pathania

**Affiliations:** 1 Department of Biosciences and Bioengineering, Indian Institute of Technology Roorkee, Roorkee, Uttarakhand, India; 2 Translational Health Science and Technology Institute, Faridabad, Haryana, India; 3 Center of Excellence in Disaster Mitigation and Management, Indian Institute of Technology Roorkee, Roorkee, Uttarakhand, India; McMaster University, Hamilton, Ontario, Canada

**Keywords:** Mn^2+^, oxidative stress, post-transcriptional regulation, small RNA, T6SS

## Abstract

Type VI secretion system (T6SS) is utilized by many Gram-negative bacteria to eliminate competing bacterial species and manipulate host cells. *Acinetobacter baumannii* ATCC 17978 utilizes T6SS at the expense of losing pAB3 plasmid to induce contact-dependent killing of competitor microbes, resulting in the loss of antibiotic resistance carried by pAB3. However, the regulatory network associated with T6SS in *A. baumannii* remains poorly understood. Here, we identified an Mn^2+^-dependent post-transcriptional regulation of T6SS mediated by a bonafide small RNA, AbsR28. *A. baumannii* utilizes MumT, an Mn^2+^-uptake inner membrane transporter, for the uptake of extracellular Mn^2+^ during oxidative stress. We demonstrate that the abundance of intracellular Mn^2+^ enables complementary base pairing of AbsR28-*tssM* mRNA (that translates to TssM, one of the vital inner membrane components of T6SS), inducing RNase E-mediated degradation of *tssM* mRNA and resulting in T6SS repression. Thus, AbsR28 mediates a crosstalk between MumT and T6SS in *A. baumannii*.

**IMPORTANCE:**

Small RNAs (sRNAs) are identified as critical components within the bacterial regulatory networks involved in fine regulation of virulence-associated factors. The sRNA-mediated regulation of type VI secretion system (T6SS) in *Acinetobacter baumannii* was unchartered. Previously, it was demonstrated that *A. baumannii* ATCC 17978 cells switch from T6− to T6+ phenotype, resulting in the loss of antibiotic resistance conferred by plasmid pAB3. Furthermore, the derivatives of pAB3 found in recent clinical isolates of *A. baumannii* harbor expanded antibiotic resistance genes and multiple determinants for virulence factors. Hence, the loss of this plasmid for T6SS activity renders *A. baumannii* T6+ cells susceptible to antibiotics and compromises their virulence. Our findings show how *A. baumannii* tends to inactivate T6SS through an sRNA-mediated regulation that relies on Mn^2+^ and retains pAB3 during infection to retain antibiotic resistance genes carried on the plasmid.

## INTRODUCTION

*Acinetobacter baumannii* is a Gram-negative pathogen and has gained importance due to its ability to cause wound and burn infections, sepsis, meningitis, urinary tract infections, bloodstream infections, and ventilator-associated pneumonia (1–5). Treating hospital-acquired *A. baumannii* infections is a major concern, as most *A. baumannii* clinical strains are multidrug resistant (6). Due to this severe global impact on public health, it is essential to identify the molecular machinery adapted by *A. baumannii* to escape from host-mediated immune response and establish pathogenesis.

One of the vital host-mediated immune responses encountered by pathogens is the phagocytic cell-mediated free metal ion limitation, termed as “host-mediated nutritional immunity” and generation of reactive oxygen species (ROS) at the site of infection (7, 8). Mn^2+^ sequestration by neutrophils at the infection site reduces the activity of bacterial metalloenzymes such as superoxide dismutase (SOD) and catalase, which bacteria use to breakdown ROS (7, 9, 10). To acquire Mn^2+^ during oxidative stress and host-mediated Mn^2+^ restriction, *A. baumannii* utilizes a high-affinity Mn^2+^ acquisition system MumT (manganese and urea metabolism transporter) (11, 12).

A synergy between the type VI secretion system (T6SS) and Mn^2+^-transporter has been observed in *Burkholderia thailandensis* (13). *B. thailandensis* secretes an effector protein TseM through T6SS that scavenges Mn^2+^ from the extracellular milieu and delivers Mn^2+^ through an outer membrane transporter (MnoT) to compensate for the scarcity of intracellular Mn^2+^ under oxidative stress (13). T6SS is one of the secretion systems frequently utilized by Gram-negative bacteria to promote contact-dependent killing of competitors by injecting harmful toxins into them (14–17). Although Mn^2+^-uptake systems have been reported as one of the major virulence factors in pathogenic bacteria (18, 19), crosstalk between Mn^2+^-transporter and T6SS remains unknown in *A. baumannii*.

The biogenesis of T6SS assembly is an enormous and energetically expensive process for bacteria (20). As a contact-dependent system with specific cellular targets (21), the expression of T6SS might be precisely regulated transcriptionally and post-transcriptionally (21, 22). Transcriptional repression of T6SS by TetR-like regulators carried by pAB3 plasmid or its derivative is observed in *A. baumannii* (23), whereas post-transcriptional regulation of T6SS has not been explored yet. The small RNAs (sRNAs) are identified as critical components within the bacterial regulatory networks involved in post-transcriptional regulation of virulence-associated factors. The sRNAs are mostly 50–500 nucleotides long, function as global regulators of numerous bacterial physiological processes, and play a crucial role in regulating several virulence factors (24, 25). The sRNAs base pair with target mRNAs and regulate the translational activity and/or the stability of that particular mRNA, often with the assistance of the RNA chaperone, Hfq (26, 27). The sRNA-mediated regulation of T6SS in *A. baumannii* remains unexplored.

In this work, we show that the breakdown in Mn^2+^-uptake by deleting *mumT* increases T6SS expression during oxidative stress. Moreover, the elevated level of intracellular Mn^2+^ by overexpressing MumT reduces T6SS expression. These observations indicate the existence of an Mn^2+^-dependent T6SS regulation in *A. baumannii*. We explored the crosstalk between MumT and T6SS with the aim of understanding the underlying mechanism of this crosstalk. Intriguingly, we identify an sRNA, AbsR28 (*A. baumannii* small RNA 28), that mediates the crosstalk between MumT and T6SS. Collectively, we show that the uptake of Mn^2+^ by MumT is necessary for AbsR28-mediated post-transcriptional regulation of *tssM* mRNA, leading to T6SS repression in *A. baumannii*.

## RESULTS

### *A. baumannii* cells that switch to T6+ are unable to withstand oxidative stress

**Fig 1:**
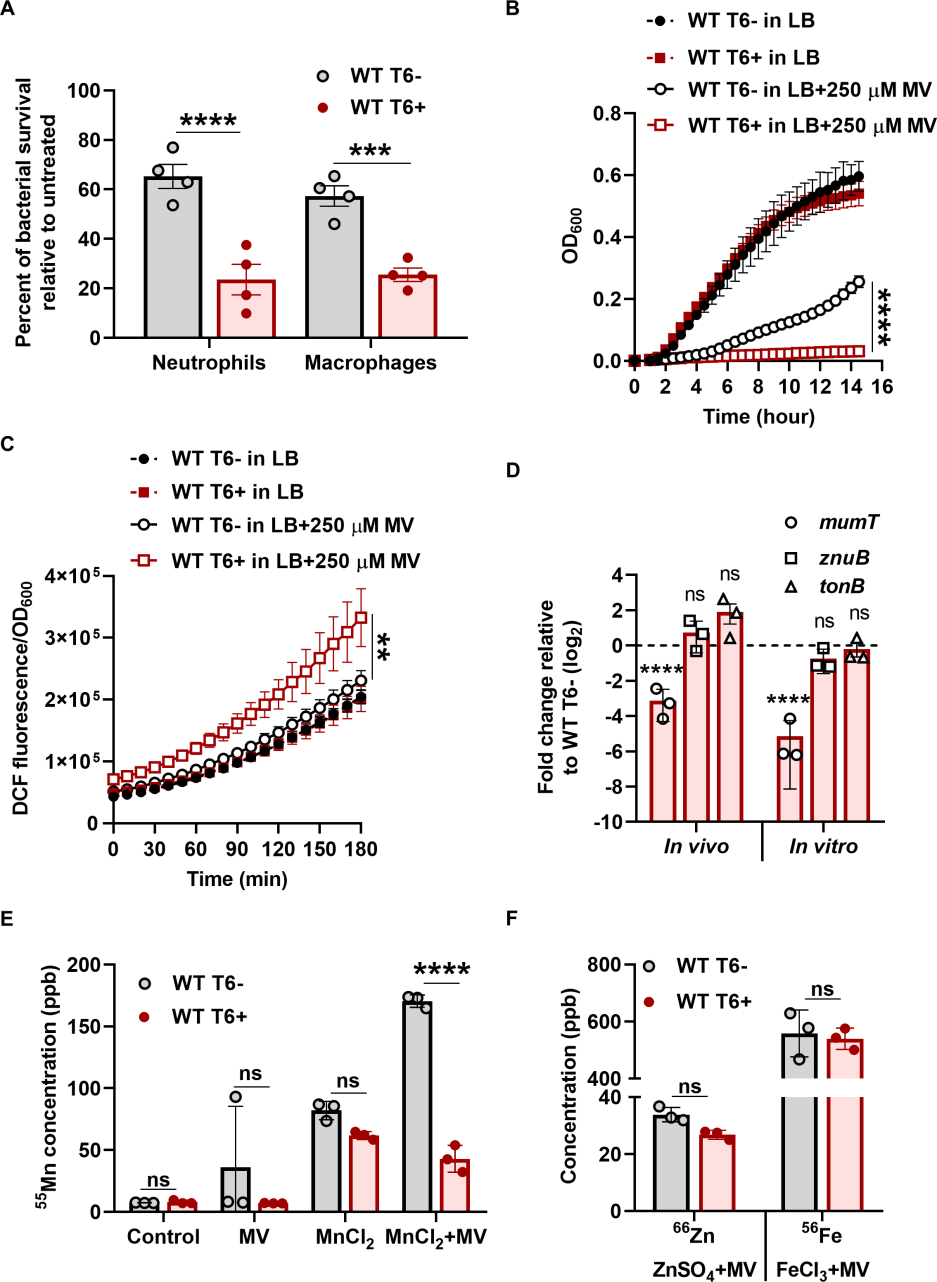
*A. baumannii* T6+ cells are defective in Mn^2+^-uptake and unable to cope with oxidative stress. (**A**) Bacterial cells were co-incubated with the phagocytic cells (human blood-derived neutrophils and macrophage RAW 264.7 cell line) for 4 h at the multiplicity of infection (MOI) of 1. The percentage of bacterial survival was enumerated by accounting for the respective untreated control (without phagocytic cells) as 100%. The data represent the mean of four independent experiments, each in biological triplicates ± SEM. (**B**) Growth of the indicated strains in the presence or absence of MV (250 µM). The data represent the mean of four biological replicates, each in technical triplicates ± SD. (**C**) Bacterial intracellular ROS generation was determined by measuring the fluorescence of DCF. The data represent the mean of four biological replicates ± SD. (**D**) Neutrophils were co-incubated with either WT T6− or WT T6+ cells, and the expression of *mumT*, *znuB*, and *tonB* in the bacterial cells was determined by quantitative reverse transcription PCR (qRT-PCR; *in vivo*). The transcript level of *mumT*, *znuB*, and *tonB* was determined in WT T6− and WT T6+ cells grown in the presence of MV by qRT-PCR (*in vitro*). The data represent the mean of three independent experiments, each in technical triplicates ± SEM. (**E and F**) Intracellular ^55^Mn, ^66^Zn, and ^56^Fe in cell pellet (OD_600_ normalized volume) were quantified by inductively coupled plasma mass spectrometry. The data represent the mean of three biological replicates ±SD. Statistical significance was determined using a multiple comparison two-way analysis of variance (ANOVA) test with the Sidak correction for multiple comparisons comparing the means of each group to one another (**A, E, and F**) and Student’s *t*-test (**B, C, and D**). ** denotes *P*-value < 0.01, *** denotes *P*-value < 0.001, **** denotes *P*-value < 0.0001, and ns denotes not significant.

The T6SS in bacteria is often found to be essential for bacterial pathogenesis as it modulates the host immune response during infection (28–31). In contrast, certain T6SSs seem to function as antivirulence mechanisms, as evidenced by increased pathogenicity in T6SS-deficient mutants relative to wild-type bacteria (32, 33). Such involvement of *A. baumannii*’s T6SS during infection remains enigmatic. Oxidative burst by phagocytic cells serves as a primary mechanism for microbial eradication in the host during infection. To assess whether *A. baumannii* cells expressing T6SS (T6+) would have an advantage in survival during phagocytic cell-mediated killing compared to *A. baumannii* cells with silent T6SS (T6−), we co-incubated phagocytic cells either with wild-type (WT-17978) T6− or WT cells that switched to T6+ cells after losing pAB3 plasmid and checked their survival. The WT T6− and WT cells that switched to T6+ cells were isolated from *A. baumannii* ATCC 17978 population based on the secretion of T6SS-associated protein Hcp (hemolysin-coregulated protein, a positive marker for expression of T6SS) using several rounds of Hcp-(enzyme-linked immunosorbent assay (ELISA) followed by western blot to detect secreted Hcp as described earlier (23). The cells that secreted Hcp were considered as T6+ (WT T6+ cells after losing pAB3 plasmid, which carries *tetR*-repressors for T6SS are referred as WT T6+ throughout the study), and the cells that showed no detectable Hcp in the cell-free supernatant were considered as T6− (WT T6− cells carrying pAB3 plasmid which carries *tetR*-repressors for T6SS are referred as WT T6− throughout the study; Fig. S1A). Surprisingly, WT T6− cells exhibited approximately 70% survival when incubated with human blood-derived neutrophils and about 60% survival with RAW 264.7 macrophages, while WT T6+ cells exhibited only around 20% and 25% survival under the same conditions, respectively (Fig. 1A). The intracellular survival of WT T6− strain was approximately 50%, while WT T6+ cells exhibited approximately 18% survival when incubated with human blood-derived neutrophils (Fig. S1B). This observation suggests that WT T6+ cells cannot withstand phagocytic cell-mediated killing. As neutrophils induce oxidative stress by producing deleterious superoxide radicals during infection, the WT T6− and WT T6+ cells were grown in an lysogeny broth (LB) medium supplemented with methyl viologen (MV) to induce superoxide radicals (34–36), and bacterial growth was measured at the indicated time points. The strains displayed almost equal growth in LB; however, upon addition of MV, WT T6+ cells exhibited a remarkable growth defect (Fig. 1B). This reinforced our previous observation that WT T6+ cells display enhanced sensitivity to oxidative stress compared to WT T6− cells. Interestingly, there are two mixed variants of *A. baumannii* ATCC 17978; one variant has a 44 kb island in the genome, and the other is devoid of that 44 kb island (37). It was demonstrated that the presence of *katX* in AbaAL44 island confers resistance to *A. baumannii* strain carrying AbaAL44 island against H_2_O_2_-mediated oxidative stress (37). It was surprising that WT T6+ strain is sensitive to oxidative stress despite possessing the AbaAL44 island, while WT T6− strain exhibits more resistance to oxidative stress despite lacking the AbaAL44 island (Fig. S1C), suggesting that the growth defect is not dependent on AbaAL44 status. Additionally, we attempted to isolate WT T6− cells containing the AbaAL44 island and WT T6+ cells lacking the AbaAL44 island to obtain isogenic strains. However, despite multiple attempts, we could not isolate either variant from *A. baumannii* ATCC 17978 population. To obtain an isogenic mutant, we knocked out the AbaAL44 island from WT T6+ cells (referred to as WT T6+ AbaAL44 ko) and conducted all subsequent experiments using the isogenic strains. Both WT T6+ (with the AbaAL44 island) and WT T6+ AbaAL44 ko strains exhibited similar survival defects when incubated with human blood-derived neutrophils compared to WT T6− cells (Fig. S1D). No significant differences in growth were observed between the WT T6+ and WT T6+ AbaAL44 ko strains under oxidative stress (Fig. S1E). These findings using the isogenic strains further reinforce that the status of the AbaAL44 island does not influence the observed phenotype. Intracellular ROS generation via Fenton chemistry is a hallmark of oxidative stress response (38, 39). Thus, we measured intracellular ROS levels in WT T6− and WT T6+ cells by growing them in LB broth with or without MV and measured the fluorescence of 2′,7′-dichlorofluorescein (DCF), which is an indicator of intracellular ROS generation. A significant increase in ROS level was observed in WT T6+ cells compared to WT T6− cells treated with MV (Fig. 1C), suggesting that WT T6+ cells are more sensitive to oxidative stress due to higher intracellular ROS accumulation. We isolated T6+ cells of two clinical strains of *A. baumannii* (RPTC2 and RPTC3) and compared the growth of WT and T6+ cells of these isolates under oxidative stress. We observed that the T6+ strains of RPTC2 and RPTC3 showed a growth defect under oxidative stress compared to their respective WT strains (Fig. S2A and B). Like *A. baumannii* ATCC 17978, RPTC2 and RPTC3 T6+ strains are more susceptible to neutrophil-mediated killing (Fig. S2C).

### *A. baumannii* T6+ cells cannot withstand oxidative stress due to inadequate intracellular Mn^2+^

Bacteria utilize metal ion-dependent SODs (Mn/Fe SOD and Cu/Zn SOD) and catalases for breaking down intracellular ROS. Therefore, metal ion homeostasis governs the cellular ROS level. We show that the abundance of intracellular Mn^2+^ is crucial for *A. baumannii*’s SOD and catalase activity (Fig. S3A and B). *A. baumannii* employs specialized metal uptake systems to import metal ions for the activity of these metalloenzymes during oxidative stress and to survive against host-mediated metal limitation (12, 40–42). Due to the compromised survival of WT T6+ cells in phagocytic cell-mediated killing, we hypothesized that WT T6+ cells may be defective in the uptake of free metal ions, which serve as cofactors for SODs and catalases required to breakdown ROS. To test this, we co-incubated neutrophils with either WT T6− or WT T6+ cells and checked the expression of *mumT*, *znuB*, and *tonB* genes involved in Mn^2+^-uptake, Zn^2+^-uptake, and Fe^2+/3+^-uptake, respectively, by quantitative reverse transcription PCR (qRT-PCR). There was no significant difference in the expression of *znuB* and *tonB* genes, but in the case of *mumT*, a ~3.5-log_2_ fold reduction was observed in WT T6+ cells as compared to WT T6− cells *in vivo* (Fig. 1D). Similarly, there was a significant fold reduction of *mumT* transcript levels in WT T6+ cells compared to WT T6− cells during oxidative stress when the bacterial cells were grown LB-medium supplemented with MV *in vitro* (Fig. 1D). Furthermore, the expression of *mumT* was downregulated in both WT T6+ and WT T6+ AbaAL44 ko strains relative to WT T6− cells, both *in vivo* and *in vitro* (Fig. S3C). Due to the fold reduction of *mumT* expression in WT T6+ cells, we hypothesized that this could lead to intracellular Mn^2+^ deficiency in WT T6+ cells under oxidative stress. To examine this, we measured the concentration of intracellular Mn^2+^ using inductively coupled plasma mass spectrometry. To determine the intracellular concentration of a specific metal ion, minimal media were preferred over complex media in this assay. The WT T6+ cells had similar to threefold lower Mn^2+^ levels in cell pellets than WT T6− cells in the presence of MnCl_2_ + MV (Fig. 1E). In contrast, there were no significant differences in Zn^2+^ and Fe^2+/3+^ levels in cell pellets of WT T6+ cells as compared to the WT T6− cells in the presence of ZnSO_4_ + MV and FeCl_3_ + MV, respectively (Fig. 1F). Taken together, these data suggest that WT T6+ cells display impaired growth under oxidative stress due to inadequate intracellular Mn^2+^, which is crucial for *A. baumannii*’s SOD and catalase activity.

### pAB3 plasmid has no role in mitigating oxidative stress

As WT T6− cells of *A. baumannii* ATCC 17978 switch to WT T6+ cells upon losing pAB3 plasmid (23), which encodes several transcription factor regulators, we speculated that the sensitivity of WT T6+ cells to oxidative stress due to downregulation of *mumT* might be a consequence of this plasmid loss, particularly since T6SS structural genes are present in both WT T6− and WT T6+ cells. To investigate this, we transformed pAB3 into the WT T6+ competent cells (which were devoid of pAB3), confirmed the transformants (Fig. S4A and B), and assessed the growth of WT T6−, WT T6+, and WT T6+ cells transformed with pAB3 (referred as WT T6+/pAB3 throughout the study) strains in LB supplemented with MV. We observed that the transformation of pAB3 does not restore the growth defect of WT T6+ cells under oxidative stress, as there is no growth difference between WT T6+ and WT T6+/pAB3 cells (Fig. S4C). Next, we sought to determine whether *mumT* expression is reduced by T6SS functionality, rendering the T6+ cells defective in Mn^2+^ uptake (scenario 1), or if the opposite is true and a breakdown in Mn^2+^ uptake results in the T6+ phenotype (scenario 2) (Fig. S4D). Consistent with the previous observation, we also observed that T6SS expression is repressed in WT T6+ strain upon the transformation of pAB3, which contains *tetR*-repressors (Fig. S4E), whereas the downregulation of *mumT* is unaffected in comparison to WT T6+ cells by the transformation of pAB3 into the WT T6+ cells (WT T6+/pAB3; Fig. S4F). This led us to infer that alteration of T6SS status does not impact *mumT* expression and ruling out the possibility of scenario 1.

### Breakdown in Mn^2+^-uptake results in an increase in T6SS expression

**Fig 2:**
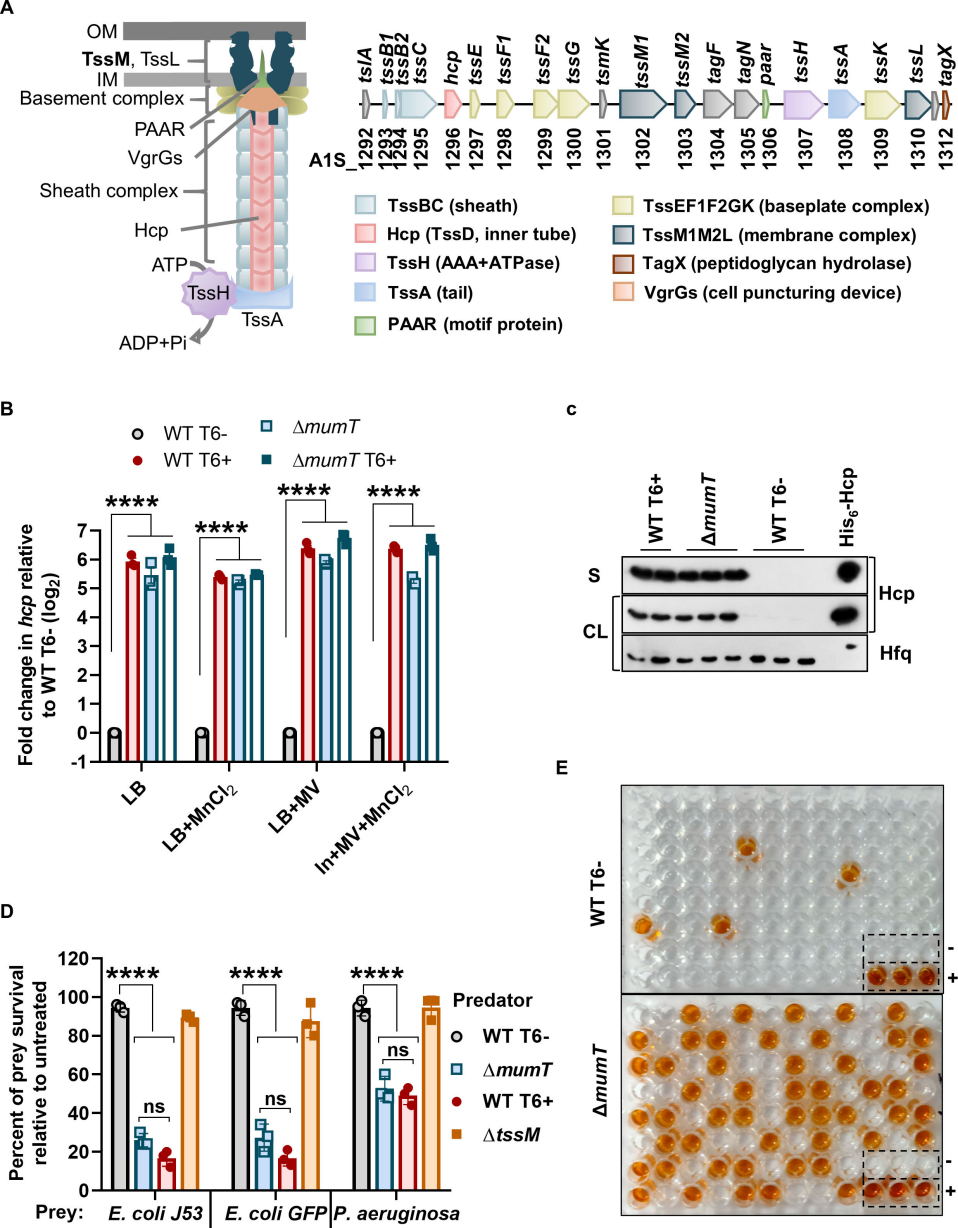
Deletion of *mumT* exhibited a significant increase in *hcp* expression under oxidative stress. (**A**) A schematic representation of T6SS and a schematic layout of the T6SS gene cluster in *A. baumannii* ATCC 17978. (**B**) Transcription of *hcp* was determined by qRT-PCR. The data represent the mean of three independent experiments, each in technical triplicates ± SEM. Statistical significance was determined using a multiple comparison two-way ANOVA test with the Sidak correction for multiple comparisons comparing the means of each group to one another. (**C**) The Hcp secretion profile of the indicated strains was confirmed by a western blot of CL and culture supernatants (**S**). WT T6+ cells and purified His_6_-Hcp were used as a positive control for this assay. The Hfq antibody was used as a loading control for CL. The data represent three independent experiments where the samples were pooled and run on one gel. (**D**) In the T6SS competition assay, prey cells were subjected to killing by incubation with WT T6−, WT T6+, Δ*mumT*, and Δ*tssM* cells. The survival percentage of prey cells was enumerated by accounting for the respective untreated control (without predator/killer cells) as 100%. WT T6+ and Δ*tssM* (the deletion mutant of TssM in WT-17978, which does not have functional T6SS) cells were used as T6+ and T6−, respectively, control for this assay. The data represent the mean of three biological replicates ±SD. Statistical significance was determined using two-way ANOVA with Tukey’s multiple comparison test for each sample with the same prey killed by the killer strains. (**E**) Detection of Hcp secretion from the cell-free supernatant of individual colonies of WT T6− and Δ*mumT* strains by Hcp-ELISA. The wells containing Hcp protein are denoted by + (positive control), and wells containing only medium are denoted as – (negative control). **** denotes *P*-value < 0.0001.

*A. baumannii* utilizes an inner membrane transporter, MumT, for Mn^2+^ uptake during host-mediated metal restriction (12). To assess whether intracellular Mn^2+^ impacts T6SS regulation (scenario 2; Fig. S4D), we first checked the effect of *mumT* on T6SS expression. To evaluate this, we created a deletion mutant of *mumT* in *A. baumannii* ATCC 17978 containing pAB3 plasmid (referred as Δ*mumT* throughout the study), grew both the WT T6− and Δ*mumT* cells in LB medium supplemented with MV and MnCl_2_, and checked the expression of *hcp* by qRT-PCR. Hcp is a structural component of T6SS (Fig. 2A). Interestingly, we observed a ~6-log_2_ fold increase in *hcp* expression in Δ*mumT* cells with respect to WT T6− cells (Fig. 2B). The data were further validated by Hcp-western blot, where Hcp expression was highly induced in Δ*mumT* cells compared to WT T6− cells in both cell lysate (CL) and cell-free supernatant (S) (Fig. 2C). We used a prey–predator assay to assess the T6SS-mediated killing of prey cells by WT T6− and Δ*mumT* cells. The killing of prey cells confirmed the expression of T6SS. As expected, Δ*mumT* cells exhibited an efficient increase in the killing of prey cells compared to WT T6− cells (Fig. 2D; Fig. S5A). We overexpressed *mumT* into WT T6+ and Δ*mumT* strains, which are defective in Mn^2+^ uptake. We observed that the elevated intracellular Mn^2+^ (Fig. S5B) due to an increase in MumT expression (Fig. S5C) remarkably reduced *hcp* expression (Fig. S5D) as well as Hcp secretion (Fig. S5E). The data were further validated using a prey−predator assay, which exhibited increased prey cell survival due to reduced T6SS activity in the complemented strains compared to their respective controls (Fig. S5F). The percentage of Hcp-secretion positive cells increased to ~50% of the entire population in Δ*mumT* strain, while only ~5% of WT T6− cells were positive under oxidative stress supplemented with MnCl_2_ (Fig. 2E). We then investigated the presence of the pAB3 plasmid in the Hcp-secretion positive Δ*mumT* cells, which were confirmed to be T6+ despite containing the pAB3 plasmid (Fig. S6A). Taken together, these data suggest that deletion of MumT results in an increase in T6SS expression. Thus, MumT seems to negatively impact T6SS regulation in *A. baumannii* under oxidative stress (scenario 2).

### AbsR28 mediates the crosstalk between *mumT* and T6SS

**Fig 3:**
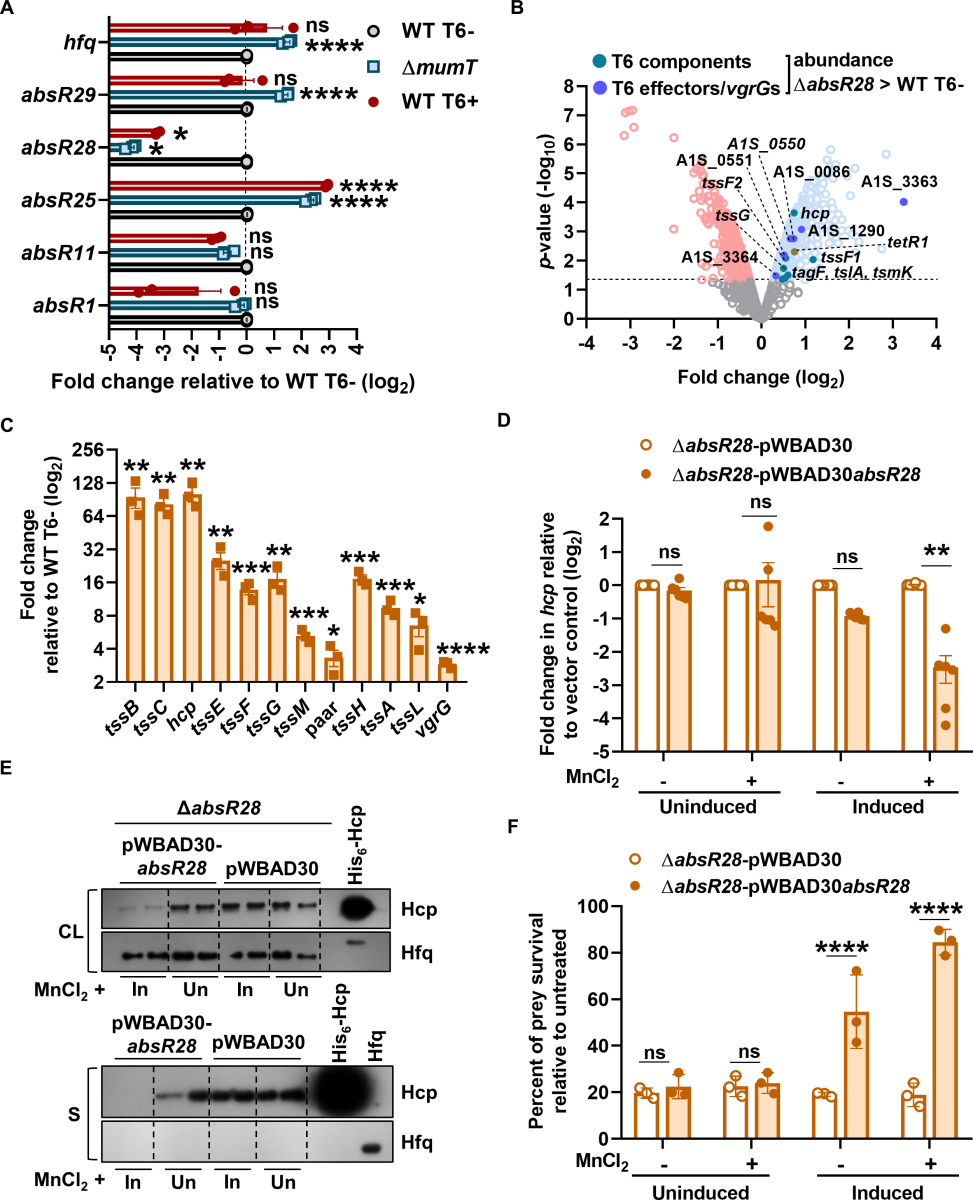
AbsR28 effectively represses T6SS in the presence of Mn^2+^. (**A**) The expression of several sRNAs and *hfq* was examined by qRT-PCR. The data represent the mean of three independent experiments, each in technical triplicates ± SEM. (**B**) RNA-seq analysis comparing RNA from MV and MnCl_2_ (both at 250 µM final conc.) treated Δ*absR28* to a WT T6− control in duplicates. A dotted black line denotes *P* < 0.05. (**C**) The transcription level of each T6SS structural gene in WT T6− and Δ*absR28* cells was determined by qRT-PCR. The data represent the mean of three independent experiments, each in technical triplicates ± SEM. (**D**) Transcription of *hcp* was determined by qRT-PCR. Uninduced denotes without arabinose, and induced denotes with arabinose. The data represent the mean of three independent experiments, each in technical triplicates ± SEM. (**E**) The Hcp secretion profile of the bacterial strains (in biological duplicates) was tested by western blot of CL and cell-free supernatant (S). The Hfq antibody was used as a loading control for CL and to confirm that the supernatants were free from the bacterial cell. Un and In denote uninduced (without arabinose) and induced (with arabinose), respectively. (**F**) In the T6SS competition assay, *Escherichia coli* J53 prey cells were subjected to killing by Δ*absR28*-pWBAD30*absR28* and Δ*absR28*-pWBAD30 strains. The data represent the mean of three biological replicates ± SD. * denotes *P*-value < 0.05, ** denotes *P*-value < 0.01, *** denotes *P*-value < 0.001, **** denotes *P*-value < 0.0001, and ns denotes not significant.

We wondered how a metal transporter could impact the bacterial secretion system. Our observation that Δ*mumT* cells are predominant in T6+ despite having *tetR1* and *tetR2* (Fig. S6A), which are transcriptional repressors of T6SS, suggests the presence of a MumT-dependent regulation of T6SS in *A. baumannii*. As the deletion of *mumT* in *A. baumannii* mimics an Mn^2+^-depleted stress condition (11), we speculated that the deletion of *mumT* might disrupt some transcriptional or post-transcriptional regulation of T6SS that causes T6SS upregulation in the Δ*mumT* cells. To begin with this hypothesis, we first checked the expression of several transcriptional regulators (i.e., *oxyR*, *zur*, and *fur*), which are known to regulate T6SS in other bacteria (13, 43, 44) at the transcriptional level in WT T6−, WT T6+, and Δ*mumT* cells grown under oxidative stress supplemented with MnCl_2_ using qRT-PCR. We also checked the transcript level of *mumR*, a transcriptional regulator of *mumT* in *A. baumannii* (12). None of the tested transcriptional regulators displayed any significant changes in expression (Fig. S6B). sRNAs play a vital role in post-transcriptional gene regulation in bacteria. The sRNA-mediated post-transcriptional regulation of T6SS is observed in *Pseudomonas aeruginosa* (45–47). In light of this, we performed a CopraRNA search (48) for all 31 putative sRNAs in *A. baumannii* that were predicted earlier using bioinformatic analysis by our lab (49), and 18 of these sRNAs showed T6SS as a putative target (Table S1). To test the hypothesis further, we first picked four sRNAs (AbsR1, AbsR11, AbsR25, and AbsR28) out of those 18 whose presence was already confirmed in *A. baumannii* and whose sequences were RACE mapped in our earlier studies (49). We checked the expression of four validated sRNAs (i.e., AbsR1, AbsR11, AbsR25, and AbsR28), and AbsR29 (which was not validated earlier to characterize it for its function), and *hfq* at the transcriptional level in WT T6−, WT T6+, and Δ*mumT* cells grown under oxidative stress supplemented with MnCl_2_ using qRT-PCR. Intriguingly, the expression of one sRNA, AbsR28, displayed a notable fold reduction in the WT T6+ and Δ*mumT* cells compared with WT T6− cells (Fig. 3A; Fig. S6C). Due to the maximum fold increase at the transcription level in the presence of MnCl_2_ under oxidative stress (Fig. S6D) and significant fold reduction in WT T6+ cells and Δ*mumT* cells, which are defective in Mn^2+^ utilization, we focused our study on evaluating the role of AbsR28 in *A. baumannii*’s T6SS regulation. We created a deletion mutant of AbsR28 in *A. baumannii* ATCC 17978 containing pAB3 plasmid (referred to as Δ*absR28* throughout the study) and assessed the growth of WT and Δ*absR28* strains in LB. Both the strains displayed an equal growth (Fig. S7A), and we confirmed that the deletion had no polar effect on the transcript abundance of A1S_2828 and A1S_2839 genes, which are immediate upstream and downstream of AbsR28, respectively (Fig. S7B). To identify the genes regulated by AbsR28, we performed RNA-seq analysis comparing the relative abundance of total mRNA transcripts of WT T6− and Δ*absR28* cells (upon exposure to oxidative stress and supplemented with MnCl_2_). In a comparison of WT T6− and Δ*absR28* strains, the expression of seven structural genes of the T6SS (*tslA* [A1S_1292]; *hcp* [A1S_1296]; *tssF1* [A1S_1298]; *tssF2* [A1S_1299]; *tssG* [A1S_1300]; *tsmK* [1301]; and *tsgF* [A1S_1304]) and six genes encoding VgrGs/T6SS effector molecules (A1S_0086, A1S_0550, A1S_0551, A1S_1290, and A1S_3363) displayed a significant fold induction in Δ*absR28* strain (Fig. 3B). All the genes encoding for functional T6SS displayed a significant fold increase in Δ*absR28* cells compared to WT T6− cells grown under oxidative stress supplemented with MnCl_2_, indicating that AbsR28 negatively regulates T6SS (Fig. 3C). Like Δ*mumT* cells, Δ*absR28* cells are also predominant in T6+ despite having *tetR1* and *tetR2* (Fig. S7C). Next, we investigated whether AbsR28 regulates T6SS in *A. baumannii* and if there is a role of Mn^2+^ in this regulation. To evaluate this, we cloned *absR28* under the control of an arabinose inducible promoter in the pWBAD30 vector and complemented the Δ*absR28* strain. We observed that the elevated level of AbsR28 due to arabinose pulse decreased the expression of Hcp, and the expression was further reduced when grown in the presence of MnCl_2_ in Δ*absR28*-pWBAD30*absR28* strain (Fig. 3D). The data were further validated by Hcp-western blot to detect secreted Hcp and a prey−predator assay, where increased expression of AbsR28 resulted in a notable reduction in Hcp secretion and increase in prey cell survival, respectively (Fig. 3E and F). These data indicate that AbsR28 efficiently represses T6SS expression in *A. baumannii* under oxidative stress in the presence of Mn^2+^.

### The binding of Mn^2+^ to AbsR28 alters its native structure

**Fig 4:**
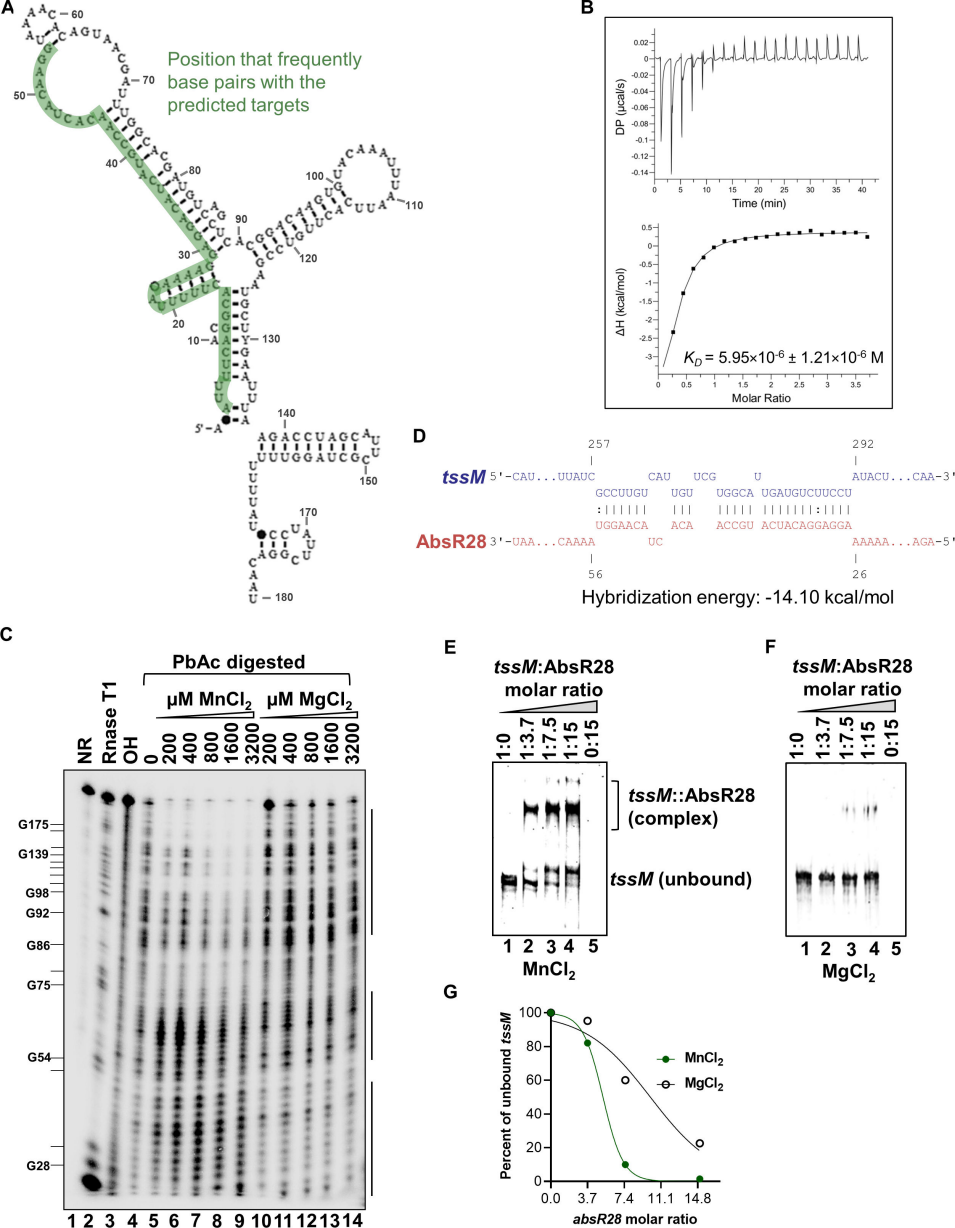
Mn^2+^ binds to AbsR28 and alters its structure. (**A**) The secondary structure of *A. baumannii* AbsR28 sRNA, predicted by Rfam software. (**B**) ITC of Mn^2+^ as ligand at an increasing concentration to AbsR28 at a fixed concentration. Molar ratio denotes ligand concentration/AbsR28 concentration in the sample cell. (**C**) Lead acetate (PbAc) probing of the 5′-end-labeled [γ-^32^P]ATP AbsR28 in an increasing concentration of either MnCl_2_ or MgCl_2_ provided in the structure buffer. Lanes indicated as T1 and OH ladders were obtained from the same labeled AbsR28 after incubation with RNase T1 and hydroxyl anions, respectively. RNase T1 digestion was performed in duplicates at 0.1 and 1.0 U concentrations. The position of cleaved G residues is marked at the left of the gel. The regions of AbsR28 that exhibit Mn²^+^-dependent changes are indicated by a solid line. (**D**) Predicted base pairing between *tssM* mRNA and AbsR28 by the CopraRNA bioinformatics tool. (**E and F**) Gel retardation assay of unlabeled *tssM in vitro* transcripts and unlabeled full-length AbsR28 *in vitro* transcripts in structure buffer containing either 10 mM MnCl_2_ or MgCl_2_. (**G**) Using ImageJ software, unbound *tssM* transcripts were quantified from panels B and C (*n* = 2 independent experiments). Nonlinear regression was used to fit the curve.

An sRNA’s ability to target a certain mRNA is contingent on its ability to form a base-pairing interaction with that mRNA at a specific location, known as the seed region (50). In AbsR28, the region that forms the most frequent base pairs with the predicted targets is positioned ~1–50 nt from the start site, making it inaccessible for interaction due to the stem formation (Fig. 4A). Due to this, we hypothesized that Mn^2+^ might bind to AbsR28 and alter its native structure, which might be essential for base pairing and its regulatory function. The dissociation constant (*K*_*D*_) between Mn^2+^ and AbsR28 was 5.95 × 10^−6^ ± 1.21 × 10^−6^ M, as measured by isothermal titration calorimetry (ITC; Fig. 4B). The affinity to Mn^2+^ is more efficient than the controls where the binding of another small RNA (i.e., AbsR25) with Mn^2+^ and AbsR28 with Mg^2+^ were assessed (Fig. S8A and B). Mutations in AbsR28 (U_89_, A_91_U_94_A_95_, and U_84_U_85_) reduced its affinity to Mn^2+^ except one mutant (A_76_A_77_; Fig. S8C). To determine whether the binding of Mn^2+^ affects the native structure of AbsR28, we performed *in vitro* structural probing with 5′-end-labeled [γ-^32^P]ATP AbsR28 using lead(II) acetate (PbAc). The Mn^2+^ concentration-dependent changes in the cleavage of AbsR28 were observed in a denaturing-PAGE, which were not very evident in the presence of Mg^2+^, used as a control (Fig. 4C). These data confirm that Mn^2+^ binds to AbsR28 and alters its native structure.

### Mn^2+^ is required for AbsR28 to interact with *tssM* mRNA

**Fig 5:**
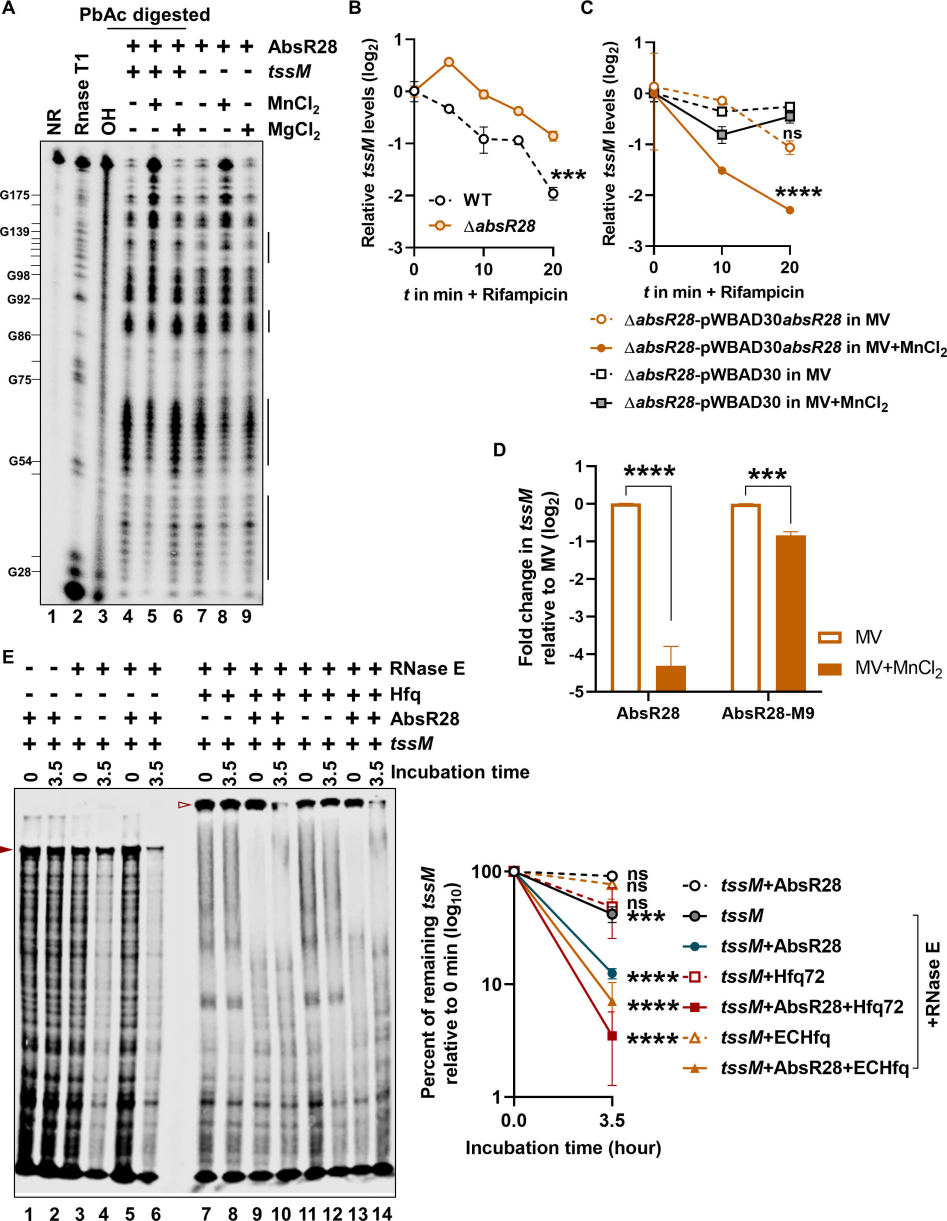
Mn^2+^ is required for AbsR28 to base pair with *tssM* mRNA and represses T6SS by potentiating RNase E-mediated degradation of *tssM* mRNA. (**A**) Lead acetate (PbAc) probing of the 5′-end-labeled [γ-^32^P]ATP AbsR28 in the presence or absence of *tssM* transcripts with either 10 mM MnCl_2_ or MgCl_2_ provided in the structured buffer. Lanes indicated as T1 and OH ladders were obtained from the same labeled AbsR28 after incubation with RNase T1 and hydroxyl anions, respectively. The position of cleaved G residues is marked at the left of the gel. The regions of AbsR28, which displayed change in PbAc-mediated cleavage due to base pairing with *tssM* transcripts, are indicated by solid lines. (**B**) The WT and Δ*absR28* cells were grown in LB supplemented with MV + MnCl_2_. The cultures were washed and treated with rifampicin. (**C**) *A. baumannii* Δ*absR28* cells harboring either plasmid pWBAD30 or pWBAD30-*absR28* were cultured to an OD_600_ of 1.0. L-arabinose was subsequently added to induce the expression of *absR28*. Following 15 minutes of induction, the cultures were washed and treated with rifampicin. For both B and C, RNA was extracted from the cells before treatment and at specified time points post-treatment. The abundance of *tssM* mRNA was quantified using qRT-PCR. The signal at the 0-minute time point was normalized to 1, and the relative expression of mRNA remaining at each time point was plotted on the *y*-axis against time on the *x*-axis. (**D**) The relative level of *tssM* transcripts under the arabinose pulse was determined by qRT-PCR. The data represent the mean ± SD. (**E**) The cleavage of the 5′-end-labeled [γ-^32^P]ATP *tssM in vitro* transcripts was assessed by incubating with RNase E in structure buffer containing MnCl_2_ for 0 and 3.5 h in the presence of either unlabeled AbsR28 (lanes 5 and 6) or *A. baumannii* Hfq72 (lanes 7 and 8) or unlabeled AbsR28 + *A. baumannii* Hfq72 (lanes 9 and 10) or *Escherichia coli* Hfq (lanes 11 and 12) or unlabeled AbsR28 + *E. coli* hfq (lanes 13 and 14). The addition of Hfq results in a slower migration which is evident from the retardation of bands (7–14). The right panel represents the quantification of the complete 5′-labeled *tssM* transcripts that remain after RNase E-mediated cleavage (denoted by a filled triangle from lanes 1–6 and an empty triangle from lanes 7–14) using ImageJ software (*n* = 3 independent experiments). Statistical significance was determined using the Student’s *t*-test (**B and E**), one-way ANOVA test with Dunnett’s multiple comparisons (**C**), and multiple comparison two-way ANOVA test with the Sidak correction for multiple comparisons, comparing the means of each group to one another (**D**). *** denotes *P*-value < 0.001, **** denotes *P*-value < 0.0001, and ns denotes not significant.

sRNAs regulate gene expression by directly base pairing with their target mRNA (51). Since T6SS in *A. baumannii* is a large gene cluster (52), we used the CopraRNA bioinformatic tool to predict the targets of AbsR28 in *A. baumannii*. A CopraRNA search using the whole-genome sequence of *A. baumannii* ATCC 17978 and full-length AbsR28 (RACE mapped sequence) as inputs predicted base pairing between *tssM* mRNA positions 258–291 and 27–55 of AbsR28 with a predicted hybridization energy value of −14.10 kcal/mol (Fig. 4D). To validate the direct interaction between AbsR28 and *tssM* mRNA, an *in vitro* RNA-RNA interaction was assessed. The addition of full-length AbsR28 at increasing concentrations to *tssM in vitro* transcripts resulted in the retardation of *tssM* transcripts in a native PAGE in the presence of Mn^2+^ (Fig. 4E); in contrast, the retardation of *tssM* transcripts was very weak in the presence of Mg^2+^, used as a control (Fig. 4F and G). The addition of full-length AbsR28 at increasing concentrations to an unrelated mRNA *pntB in vitro* transcripts (used as a negative control) did not show any retardation (Fig. S9A). Furthermore, our in-line probing of AbsR28 revealed that the seed region of AbsR28 was protected from cleavage when *tssM* transcripts and Mn^2+^ were present, indicating a potential interaction between the two RNAs in the presence of Mn^2+^ (Fig. 5A). However, the changes in the cleavage toward the 3'-region of AbsR28 are more pronounced in the same condition indicating the structure alteration after base pairing. For further validation of AbsR28-*tssM* binding, AbsR28 was mutated at the seed region (AbsR28-M9; A_27_G_28_G_29_A_30_ was mutated to U_27_C_28_C_29_U_30_; Fig. S9B). The retardation of *tssM* transcripts was much weaker even with higher concentrations of AbsR28-M9 compared to native AbsR28 (Fig. S9C and D). Furthermore, we show that the compensatory mutation in *tssM* mRNA (tssM*) restored the binding of AbsR28-M9 (Fig. S9E). Collectively, these findings indicate that the direct interaction of AbsR28 and *tssM* transcripts is Mn^2+^ dependent. Additionally, we extended our investigation into the conservation of AbsR28-*tssM* base pairing across various *Acinetobacter* species using CopraRNA analysis. This revealed a significant degree of complementary conservation among most species, except for two (Fig. S10A), similar to the observed base pairing in *A. baumannii* ATCC 17978.

### AbsR28 potentiates RNase E-mediated *tssM* mRNA degradation

To study the molecular mechanism of AbsR28-meditated T6SS repression in *A. baumannii*, we checked the effect of AbsR28-*tssM* interaction on the stability of target mRNA (i.e., *tssM*) in WT and ∆*absR28* strains. We observed a faster reduction in the stability of *tssM* mRNA *in vivo* in WT strain compared to ∆*absR28* strain after being treated with rifampicin (Fig. 5B). Furthermore, we assessed AbsR28-dependent changes in t*ssM* mRNA stability. AbsR28 was induced from the plasmid pWBAD30-*absR28* in a Δ*absR28* strain for 15 minutes, after which transcription was halted using rifampicin treatment. The decay of *tssM* mRNA was then monitored by qRT-PCR. AbsR28 markedly reduced the stability of *tssM* mRNA *in vivo* in the presence of Mn^2+^ (Fig. 5C). We observed that the elevated level of native AbsR28 reduced the level of *tssM* transcripts more significantly compared to the AbsR28-M9 via *in vivo* pulse expression assay (Fig. 5D). This result confirmed that *tssM* processing depends on the direct interaction between AbsR28 and *tssM*. sRNAs induce the decay of target mRNA by RNase E-mediated mRNA degradation in an Hfq-dependent manner and repress its translation (53–59). We performed an *in vitro* RNase E-mediated degradation assay where the 5′-end of the *in vitro* transcribed *tssM* was labeled with [γ-^32^P]ATP, incubated with purified RNase E in the presence or absence of unlabeled AbsR28 with *A. baumannii* Hfq72 or *Escherichia coli* Hfq protein. We quantified the remaining full-length *tssM* transcripts post-incubation. As anticipated, RNase E exhibited a significantly increased rate of decay for *tssM* transcripts in the presence of AbsR28 and Hfq compared to the transcripts incubated alone (Fig. 5E). These data demonstrate the molecular mechanism behind AbsR28-mediated T6SS regulation where the degradation of *tssM* mRNA by RNase E is strongly enhanced by base pairing to AbsR28 in the presence of Mn^2+^ and reduces the abundance of *tssM* transcripts for T6SS assembly.

### AbsR28 contributes to the fitness of *A. baumannii* during infection

**Fig 6:**
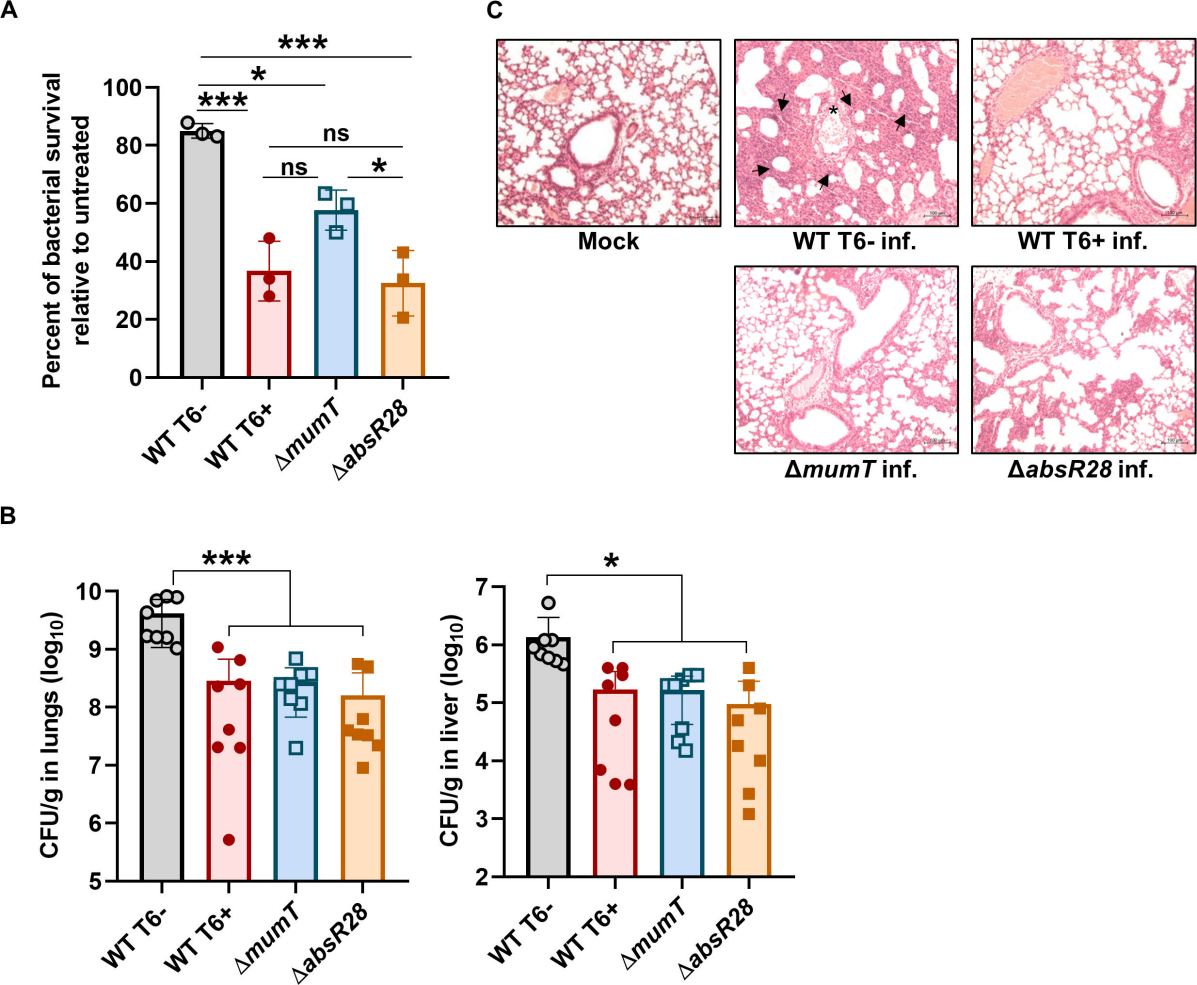
Mn^2+^-dependent AbsR28-mediated T6SS repression is required for *A. baumannii* pathogenesis. (**A**) Bacterial strains were co-incubated with neutrophils for 4 h at an MOI of 1. The percentage of bacterial survival was enumerated, accounting for the respective untreated control (without neutrophils) as 100%. The data represent the mean of three independent experiments, each in biological triplicates ± SEM. (**B**) Enumeration of bacterial burden recovered from mice lungs and liver (*n* = 8) infected with WT T6−, WT T6+, Δ*mumT*, or Δ*absR28* strains at 36 hpi. Data represent mean ± SD. (**C**) Histopathology of the mice lungs (Hematoxylin and eosin stained) infected with WT T6−, WT T6+, Δ*mumT*, or Δ*absR28* strains at 36 hpi. Tissue infiltration is indicated by necrosis (arrowheads) and alveolar inflammation (asterisks). The scale bar is 100 µM. Statistical significance was determined using the one-way ANOVA test with Tukey’s multiple comparisons (**A and B**). * denotes *P*-value < 0.05, *** denotes *P*-value < 0.001, and ns denotes not significant.

In addition to T6SS regulation, we observed that the deletion of AbsR28 primarily affects genes involved in bacterial metabolism (carbohydrate, phenylalanine, cysteine, and methionine metabolism) and stress response (two-component system and ABC transporters) in RNA-seq data (Table S2). Since AbsR28 remains uncharacterized in *A. baumannii* pathophysiology, we aimed to explore the importance of AbsR28-mediated gene regulation in the pathogenesis of *A. baumannii*. We co-incubated human blood-derived neutrophils with either WT T6−, WT T6+, Δ*mumT*, or Δ*absR28* cells and checked their survival. WT T6− cells exhibited ~85% survival, whereas Δ*absR28* cells showed only ~40% survival against neutrophil-mediated killing compared to untreated cells (Fig. 6A). We next assessed the pathogenesis of WT T6−, WT T6+, Δ*mumT*, and Δ*absR28* strains in a mouse model of *A. baumannii*-induced pneumonia where the strains were intranasally administered to BALB/c mice. In consistency with the previous observation that the deletion of *mumT* in *A. baumannii* results in compromised survival in mice due to a defect in Mn^2+^-uptake (12), we also observed that Δ*mumT* strain had a lower organ burden compared to the WT T6− strain. Additionally, WT T6+ and Δ*absR28* strains had significantly reduced burdens in the lungs and liver when compared with the WT T6− strain (Fig. 6B). Lungs isolated from mice infected with the WT T6− strain exhibited a remarkable tissue infiltration as indicated by necrosis and alveolar inflammation, whereas the mice lungs infected with either WT T6+, Δ*mumT*, or Δ*absR28* strains did not show any notable damage (Fig. 6C). Together, the data suggest that AbsR28 contributes to *A. baumannii* fitness during infection.

## DISCUSSION

During bacterial infection, the host recruits phagocytic cells at the site of infection, which creates oxidative stress and limits the availability of free metal ions (12, 60, 61). For certain bacteria, T6SS sequesters extracellular free metal ions and helps them cope with oxidative stress and host-mediated free metal restriction, so it is advantageous for them to express T6SS during oxidative stress (13, 43, 62). However, the role of T6SS in *A. baumannii*’s ability to survive under phagocytic cell-mediated oxidative stress has not been explored yet. Unlike *B. thailandensis*, where a synergistic relationship exists between Mn^2+^-uptake system and T6SS expression (13), we found that *A. baumannii* cells that switch to T6+ cells are sensitive to oxidative stress due to inadequate intracellular Mn^2+^, which is required as a cofactor for SOD and catalase to breakdown intracellular ROS generated during oxidative stress (Fig. 1; Fig. S1 and S3). In this study, we found that the lowered transcript levels of Mn^2+^ uptake gene *mumT* to be an underlying contributor to the reduced Mn^2+^ levels within the T6+ cells. Counterintuitively, we inferred that the loss of pAB3 plasmid, which harbors TetR-like repressors, is not responsible for this phenomenon. This observation was validated through the re-introduction of pAB3 plasmid which failed to restore *mumT* levels akin to that of the wild type. Hence, we found it imperative to uncover the probable cause for this downregulation of *mumT* expression by investigating whether there is a role of any other global regulator behind this effect. We first looked into the expression of MumR and Fur as MumR is known to be a positive transcriptional regulator of *mumT* in *A. baumannii* (12), and Fur-mediated transcriptional regulation of Mn^2+^ transporters has been reported in other bacteria (63). However, our comparison of transcript levels for *mumR* and *fur* in WT T6− and WT T6+ cells revealed no significant differences (Fig. S6B), suggesting that they do not play a role in the downregulation of *mumT* in WT T6+ cells. In *B. thailandensis*, OxyR regulates the expression of T6SS to enhance extracellular Mn^2+^ scavenging via TseM effector (13). OxyR is also known to regulate Mn^2+^ transporter in bacteria (64). In contrast, we found no significant changes in *oxyR* transcript levels between WT T6− and WT T6+ cells under oxidative stress (Fig. S6B). These findings indicated that T6SS might not influence the expression of *mumT*, and the lowered level of *mumT* in T6+ cells might be a cause and not a consequence of the phenotypic change resulting from T6− to T6+. Hence, we hypothesized that *mumT* may influence T6SS expression, suggesting that *A. baumannii* cells that become defective in *mumT* expression under oxidative stress switch from T6− to T6+. This points at the role of *mumT* as a phenotypic switch that leads to T6+ transition under oxidative stress. To check the impact of intracellular Mn^2+^ on T6SS expression, we observed that a breakdown in Mn^2+^-uptake promotes a significant increase in T6SS expression under oxidative stress (Fig. 2; Fig. S5), indicating at the existence of an Mn^2+^-dependent alternative regulation of T6SS apart from pAB3 in *A. baumannii* ATCC 17978. Next, we focused on determining the molecular mechanism behind this regulation by investigating other reported avenues with similar roles in different bacterial strains. The sRNA-mediated post-transcriptional regulation of T6SS is well documented in *P. aeruginosa* (45–47), which belongs to the same taxonomic order as *A. baumannii*. However, in *A. baumannii*, to date no sRNAs have been identified that regulate T6SS, which led us to explore this aspect. Through our experiment, we were able to ascertain the role of the sRNA AbsR28 in mediating a crosstalk between MumT and T6SS during oxidative stress in *A. baumannii*. We show that binding of Mn^2+^ to AbsR28 alters its native structure and results in a complementary base pairing with *tssM* transcripts (Fig. 4). The exact Mn^2+^-binding region of AbsR28 remains to be determined. TssM is an indispensable structural component of the T6SS membrane complex which is essential for T6SS assembly, and its deletion disrupts the functionality of T6SS in *A. baumannii* (65–69). Given the inverse relationship between Mn^2+^ levels and T6SS expression, we looked into whether Mn^2+^ drives the sRNA-mediated regulation of T6SS. We observed that the stability of *tssM* transcripts was significantly reduced when AbsR28 was pulse expressed in the presence of Mn^2+^ (Fig. 5C). The role of AbsR28 was further confirmed through our in-line probing assay which demonstrates that AbsR28 potentiates RNase E-mediated degradation of *tssM* transcripts with the assistance of RNA chaperone Hfq (Fig. 5E).

**Fig 7:**
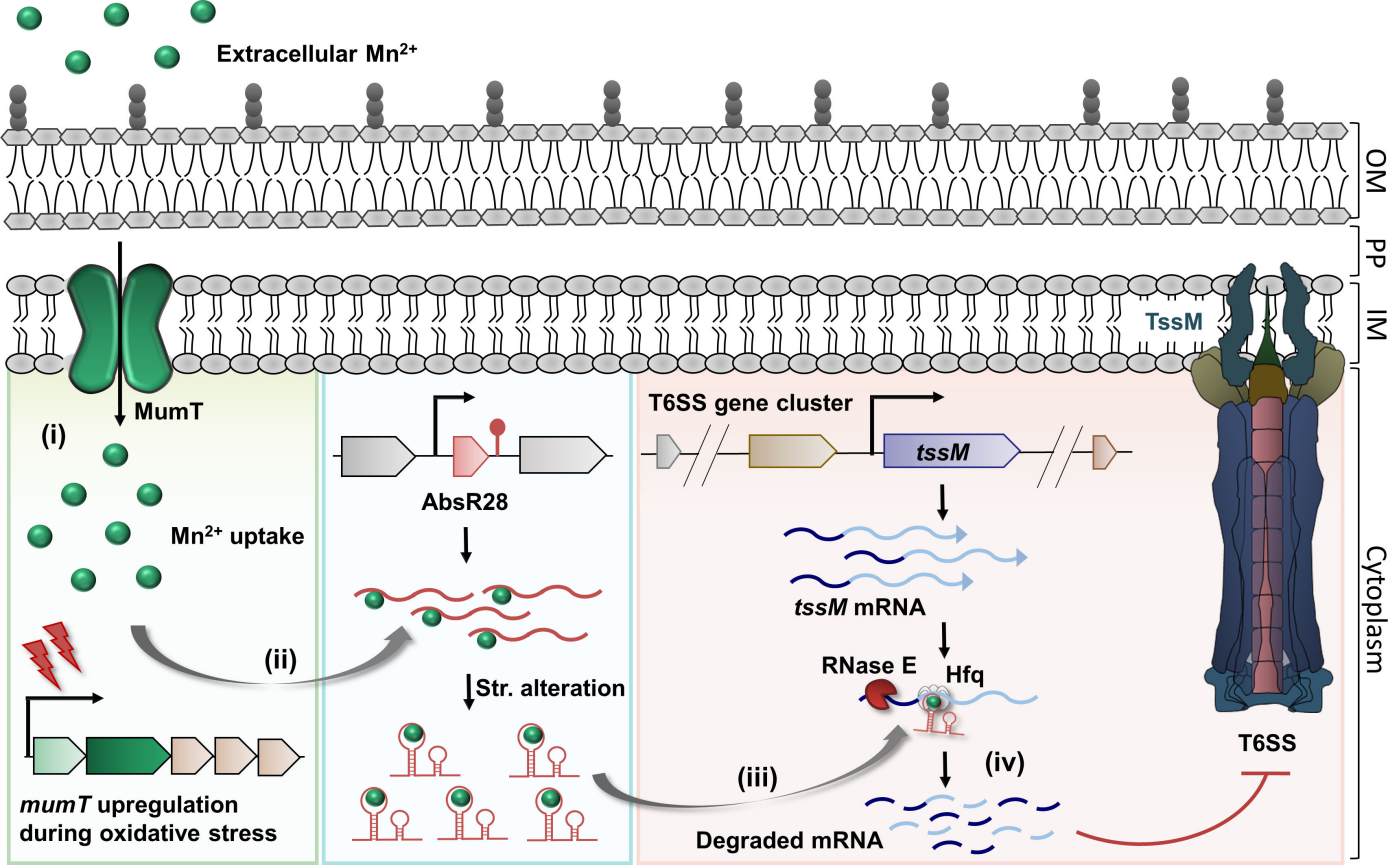
A proposed model of AbsR28-mediated post-transcriptional repression of T6SS in *A. baumannii* during oxidative stress. In the presence of Mn^2+^, AbsR28 base pairs with *tssM* mRNA and induces RNase E-mediated processing of *tssM*, which results in the repression of T6SS in *A. baumannii*.

In summary, we have explored the molecular mechanism of AbsR28-mediated T6SS repression in *A. baumannii* ATCC 17978 (Fig. 7). The sequential events of this proposed pathway are as follows: (i) during oxidative stress, *A. baumannii* cells upregulate *mumT* to increase Mn^2+^ uptake; (ii) intracellular Mn^2+^ binds to AbsR28 and alters its native structure; (iii) the altered structure of AbsR28 aids in the complementary base pairing with *tssM* mRNA transcripts and triggers RNase E processing; (iv) degradation by RNase E reduces the abundance of *tssM* transcripts and thereby represses the T6SS expression. *A. baumannii* cells that lose this controlled regulation result in T6+ phenotype. Our findings unveil a novel Mn²^+^-dependent, sRNA-mediated regulatory mechanism governing T6SS expression in *A. baumannii*. This study enhances our understanding of sRNA-mediated gene regulation and emphasizes the importance of metal ion availability in bacterial survival strategies under oxidative stress.

The T6SS is often inactive in clinical isolates of *A. baumannii* (70–72). Several factors may contribute to this: (i) T6SS expression is energetically demanding, leading to prioritization of energy for other cellular processes; (ii) T6SS components are immunogenic (73, 74), so it may be advantageous for *A. baumannii* T6SS inactive strains to evade the host-mediated immune response (75); (iii) switching from T6− to T6+ compromises antibiotic resistance associated with the plasmid pAB3 (23); (iv) T6+ isolate from urinary tract infection has been shown to exhibit reduced virulence due to loss of pAB5 (a pAB3-family member), which regulates several chromosomal virulence factors (76); (v) firing the T6SS arrow involves bacteria rupturing the outer membrane, which could potentially disrupt the membrane integrity and render *A. baumannii* T6+ cells more sensitive to oxidative stress. Therefore, *A. baumannii* may favor sRNA-mediated regulation to regulate T6SS, prioritizing antibiotic resistance and virulence. An intriguing question remains regarding the regulatory mechanisms employed by *A. baumannii*: under what conditions does this pathogen preferentially utilize sRNA-mediated regulation of the T6SS as opposed to plasmid-mediated regulation, or are these two regulatory pathways interconnected? Furthermore, our results highlight the need for future research to identify additional genes that are differentially expressed between WT T6− and WT T6+ cells under oxidative stress conditions. Understanding the dynamics between these regulatory mechanisms is crucial for elucidating the pathogen’s adaptability and virulence. Future studies should focus on a comprehensive analysis between clinical and laboratory strains to better understand T6SS regulation under varying stress conditions.

## MATERIALS AND METHODS

### Bacterial strains and growth conditions

Bacterial strains and plasmids used in this study are listed in Tables S3 and S4. A. *baumannii* strains were grown at 37°C in LB broth or LB agar. *E. coli* J53 strains were grown in LB broth or LB agar, supplemented with 100 µg/mL sodium azide when necessary. *E. coli*-pNYL GFP strains were grown in LB broth or LB agar, supplemented with 50 µg/mL kanamycin when necessary. *P. aeruginosa* PAO1 strains were grown in LB broth or LB agar. *A. baumannii* strains that contain the pWBAD30 vector were grown in LB broth or LB agar, supplemented with 50 µg/mL kanamycin.

### Estimation of cell survival from phagocytosis

The estimation of bacterial survival from phagocytic cell-mediated killing was performed as described previously (77). The detailed procedure is provided in the supplemental material.

### ROS quantification

Bacterial intracellular ROS levels were estimated using 2′,7′-dichlorofluorescein diacetate (Thermo Fisher Scientific, D399), added at a final concentration of 100 µM. The detailed procedure is provided in the supplemental material.

### Quantitative RT-PCR analysis

Human blood-derived neutrophils were co-incubated with the bacterial strains as described in the above section. The details procedure for RNA extraction and cDNA synthesis is provided in the supplemental material. The transcript abundance was calculated using the ΔΔ*C*_*T*_ method (78) and normalized by the 16S gene.

### Quantification of intracellular metal content

The concentration of metal content in bacterial cells was determined as described previously (12) with some modifications. The detailed procedure is provided in the supplemental material.

### Western blot analysis for Hcp

From mid-log phase cultures of the indicated strains, a 0.1% inoculum from the overnight (O/N) cultures was then subcultured into 5 mL of fresh LB medium containing MnCl_2_ at a final concentration of 250 µM and grown at 37°C with shaking to an OD_600_ of 0.6 (mid-log phase) for each strain. MV was added to the culture at a final concentration of 250 µM and incubated for another 2 h. The Hcp secretion phenotype of the strains was performed by Hcp-western blot as described in the supplemental material. Primary anti-Hfq-antibody raised in rabbit at a dilution of 1:1,000 was used as a loading control for CL.

### Bacterial killing assay

Bacterial killing assays were performed as described previously (23). *A. baumannii* T6− and Δ*mumT* strains were used as killers/predators, while *E. coli* J53, *E. coli*-pNYL GFP, or *P. aeruginosa* were used as prey in this assay. The detailed procedure is provided in the supplemental material.

### ELISA assay for Hcp

From the mid-log phase cultures of the indicated strains, a 0.1% inoculum from the O/N cultures was then subcultured into 5 mL of fresh LB medium containing MnCl_2_ at a final concentration of 250 µM and grown at 37°C with shaking to an OD_600_ of 0.6 (mid-log phase) for each strain. MV was added to the culture at a final concentration of 250 µM and incubated for another 2 h. The rest of the protocol for Hcp-ELISA was performed as mentioned in the supplemental material.

### RNA-sequencing data analysis

RNA was isolated from WT T6− and Δ*absR28* cells grown in LB supplemented with MnCl_2_ (250 µM) to an OD_600_ of 0.6 (mid-log phase), treated with MV (250 µM) for 2 h, and purified as described above. RNA sequencing was performed by Biokart India Pvt. Ltd. (India) using the Illumina HiSeq 4000 platform (Illumina). Analysis was performed by Rockhopper v 2.0.3. Comparative and statistical analyses were performed using iGeak v1.0a using the reference *A. baumannii* ATCC 17978 genome (National Center for Biotechnology Information [NCBI]: CP000521.1).

### Pulse expression studies

An arabinose inducible vector, pWBAD30, was modified from the pBAD30 backbone for this study. The pWBAD30-*absR28* plasmid was transformed into Δ*absR28* electrocompetent cells, and the transformants were selected on LB-agar plates containing kanamycin (50 µg/mL). Δ*absR28* transformed with pWBAD30 served as a vector control. The bacterial survival assay was performed as described in the supplemental material in the presence of arabinose (0.2% [wt/vol] final concentration).

### Isothermal calorimetry

ITC titrations were performed using 600 µM MnCl_2_ as ligand and 30 µM *in vitro* transcribed AbsR28 in the reaction cell. The detailed procedure is provided in the supplemental material.

### *In vitro* RNA transcription and 5′-end labeling

*In vitro* transcription (IVT) was performed as described earlier (79). The template for IVT was obtained using *A. baumannii* ATCC 17978 genomic DNA as a PCR template and forward primers containing the T7 promoter listed in Table S5. The detailed procedure is provided in the supplemental material.

### *In vitro* structural probing

*In vitro* structural probing was performed as described earlier (80) with some modifications using *in vitro* transcribed AbsR28 5′-labeled with [γ-^32^P]ATP. The detailed procedure is provided in the supplemental material.

### Gel retardation assay

The gel retardation assay was performed as described earlier (81) with some modifications using unlabeled *tssM in vitro* transcripts (250 nt upstream and 250 nt downstream from ATG) and full-length AbsR28 *in vitro* transcripts. The detailed procedure is provided in the supplemental material.

### RNase E-mediated degradation assay

Rnase E-mediated degradation assay was performed using the *in vitro* transcript *tssM* 5'-labeled with [γ-^32^P]ATP. The detailed procedure is provided in the supplemental material.

### Mice infection model for *A. baumannii* pneumonia

Adult (6–8-week-old) age-matched male BALB/c mice (*n* = 8 for each group, determined using G*Power analysis) were infected intranasally with *A. baumannii*, and colony-forming units (CFU) were enumerated as previously described (82). The detailed procedure is provided in the supplemental material.

### Quantification and statistical analysis

Statistical analyses were performed using GraphPad Prism 8. Band quantification from gels was performed using ImageJ and analyzed using GraphPad Prism 8. The details about the number of repeated experiments, statistical tests used, significance values, and group sizes are indicated in each figure legend.

## Data Availability

Raw RNA-sequence data have been deposited at NCBI Sequence Read Archive (NCBI SRA) with accession number PRJNA865908. Target prediction of AbsR28 was performed using CopraRNA (https://rna.informatik.uni-freiburg.de/CopraRNA, accessed on 2 October 2020) using the RACE-mapped sequence of AbsR28 (45) as input for *Acinetobacter baumannii* ATCC 17978 (NCBI reference sequences: NZ_CP018664, NZ_CP049363, NZ_CP039028, and NZ_CP039023) as an organism of interest. Any additional information required to analyze the data reported in this paper is available upon request from the corresponding author.

## SUPPLEMENTAL MATERIAL

Supplemental file mbio.03025-24-s0001.docx Additional experimental details, Fig. S1 to S9, and Table S1 to S5.

ASM does not own the copyrights to Supplemental Material that may be linked to, or accessed through, an article. The authors have granted ASM a non-exclusive, world-wide license to publish the Supplemental Material files. Please contact the corresponding author directly for reuse.
