# Supplementary material for: Acinetobacter baumannii represses type VI secretion system through a manganese-dependent small RNA-mediated regulation
Source: mBio. 2024 Dec 20;16(2):e03025-24. doi: 10.1128/mbio.03025-24 (PMC11796373; doi:10.1128/mbio.03025-24)
Supplement: Supplemental file — Additional experimental details, Fig. S1 to S9, and Table S1 to S5. [file mbio.03025-24-s0001.docx]

**SUPPLEMENTAL MATERIAL**

***Acinetobacter baumannii* represses type VI secretion system through a manganese-dependent small RNA-mediated regulation**

Somok Bhowmik, Avik Pathak, Shivam Pandey, Kuldip Devnath, Abhiroop Sett, Nishant Jyoti, Timsy Bhando, Jawed Akhter, Saurabh Chugh, Ramandeep Singh, Tarun Kumar Sharma, Ranjana Pathania^*^

^*^Address correspondence to Ranjana Pathania, ranjana.pathania@bt.iitr.ac.in

**DETAILED METHODS**

**Isolation of *A. baumannii* T6- and T6+ cells**

*A. baumannii* T6- and T6+ cells were isolated using Hcp-ELISA as described previously (1) with some modifications. Briefly, a fresh colony of wild-type *A. baumannii* ATCC 17978 streaked on LB-agar plate was used to inoculate in 5 mL of LB-medium overnight (O/N) at 37 ⁰C with shaking. 0.1% inoculum from the O/N culture was then subcultured into fresh 5 mL of LB-medium and grown at 37 ⁰C with shaking to an OD_600_ of 0.6 (mid-log phase). The culture was serially diluted in LB medium and plated on LB-agar, followed by incubation at 37 ⁰C for O/N to obtain more than 100 isolated colonies. Individual colonies were inoculated in a 96-well plate containing 200 µL of LB medium/well and incubated at 37 ⁰C for O/N with gentle shaking. After O/N growth, the plate was centrifuged to pellet down the bacterial cells and 75 µL of the supernatant from each well was transferred to a 96-well ELISA plate (Thermo Fisher Scientific) containing 25 µL of binding buffer (0.0258 M sodium carbonate, 0.0742 M sodium bicarbonate, pH 9.5) in each well (for example, A1 supernatant from the 96-well plate was transferred to A1 of 96-well ELISA plate). The ELISA plate was incubated at 4 ⁰C for O/N on a gel rocker for efficient binding. The plate was then washed with 1X PBS and blocked with 200 µL of blocking solution (5% w/v skim milk in PBST; 1X PBS containing 0.1% inoculum v/v Tween-20) for 1 h at room temperature (RT). Primary anti-Hcp-antibody raised in the rabbit at a dilution of 1:10000 in blocking solution (2.5% w/v skim milk in PBST) was used at 100 µL/well to probe at 4 ⁰C for O/N. Following three successive washes with PBST, HRP-conjugated goat anti-rabbit secondary antibody (Thermo Fisher Scientific, 31460) at a dilution of 1:20000 in a solution (1X PBS containing 0.1% inoculum v/v Tween-20) was added 100 µL/well and incubated for 1 h at RT in the dark. After three successive washes with PBST and one wash with PBS, 50 µL substrate (citrate phosphate buffer at pH 5.6 containing H_2_O_2_ and *o*-Phenylenediamine dihydrochloride) was added to each well and waited for 10-15 min to develop a yellow color. The reaction was stopped by adding 3N HCl, which will turn yellow to orange, and measured the OD at 495 nm. Purified His_6_-Hcp was used as a positive control, and unsupplemented LB medium as a negative control for the assay. A well that appears to have the T6SS+ signal (i.e., develops an orange color after ELISA) was marked and the cells from that particular well of the 96-well plate (the source of the supernatant sample) were isolated (considered at *A. baumannii* T6+ after 1^st^ round of ELISA). Similarly, a well that appears to have the T6SS- signal (i.e., does not develop any color after ELISA) was marked and the cells from that particular well of the 96-well plate (the source of the supernatant sample) were isolated (considered at *A. baumannii* T6- after 1^st^ round of ELISA). The *A. baumannii* T6- and T6+ cells from 1^st^ round ELISA were plated on LB agar, and further Hcp-ELISA (2^nd^ round) was performed using freshly isolated individual colonies to confirm the T6SS phenotype.

Further, *A. baumannii* T6+ cells were checked for Hcp secretion phenotype by Hcp-Western blot. The *A. baumannii* T6- and T6+ cells from the 2^nd^ round of ELISA were inoculated in LB medium and grew overnight (O/N) at 37 ⁰C with shaking. Individually, 0.1% inoculum from the O/N cultures was subcultured into fresh 5 mL of LB-medium and grown at 37 ⁰C with shaking to an OD_600_ of 0.6 (mid-log phase) for both strains. Bacterial cells were harvested from 1 mL cultures and supernatants were collected. The supernatants were filtered through a 0.22 µm syringe filter (Merk Millipore Ltd.) and concentrated using trichloroacetic acid. The pellets were dissolved in 1X SDS-gel loading dye and heated at 95 ⁰C for 5 min. Whole-cell lysate (OD_600_ normalized volume) and supernatants were run on a 15% SDS-PAGE for separation and transferred to a PVDF membrane (Cytiva, GE10600023). Following blocking (5% w/v skim milk in PBST; 1X PBS containing 0.1% inoculum v/v Tween-20) for 1 h at RT, the membrane was probed by primary anti-Hcp-antibody raised in the rabbit at a dilution of 1:1000 in blocking solution (2.5% w/v skim milk in PBST) at 4 ⁰C for O/N on a gel rocker. Following five successive washes with PBST, HRP-conjugated goat anti-rabbit secondary antibody (Thermo Fisher Scientific, 31460) at a dilution of 1:20000 in a solution (1X PBS containing 0.1% inoculum v/v Tween-20) was added and incubated for 1 h at RT in the dark. After five successive washes with PBST and one wash with PBS, ECL substrate (TakaRa) was added and developed onto an X-ray film.

After isolating WT T6- and WT T6+ variants from the wild-type strain *A. baumannii* ATCC 17978 by Hcp-ELISA and validation by Hcp-Western blot, glycerol stocks were prepared. The WT T6- and WT T6+ variants were freshly streaked, and the phenotype was confirmed by several Hcp-ELISA assays. Before and after each experiment, the Hcp-secretion profiles of the WT T6- and WT T6+ strains were checked by Western blot to confirm the phenotypes.

**Estimation of cell survival from phagocytosis**

All experiments using human blood-derived neutrophils under protocol BT/IHEC-612020/7865 were reviewed and approved by the Institute Human Ethics Committee (HEC) of the Indian Institute of Technology Roorkee. Neutrophils were isolated from human blood using Polymorphprep (ProteoGenix) according to manufacturer’s instruction and diluted to obtain a final concentration of 1x10^4^ cells/well. A freshly streaked colony of WT T6- and WT T6+ strain on LB-agar plates were used to inoculate in 5 mL of LB-medium for O/N at 37 ⁰C with shaking. 0.1% inoculum from the O/N cultures was then subcultured into fresh 5 mL of LB-medium and grown at 37 ⁰C with shaking to an OD_600_ of 0.6 (mid-log phase). Bacterial cells were harvested and diluted to obtain 10^4^ CFU/µL. The diluted bacterial cultures were then opsonized in fetal bovine serum (non-heat treated) for 15 min. Neutrophils were then co-incubated with bacterial strains at an MOI of 1:1 ratio in RPMI 1640 cell culture medium (HIMEDIA, AL028A) and incubated at 37 ⁰C in an animal tissue culture incubator (Eppendorf). The same method was performed for the macrophage RAW 264.7 cell line in the DMEM medium (HIMEDIA, AL007A). After 4 h of infection, the medium supernatants were serially diluted and plated onto Leeds *Acinetobacter* medium plates. After incubation at 37 ⁰C for O/N, the bacterial colonies were enumerated and the percent growth was quantified by dividing the CFU of the particular *A. baumannii* strain-neutrophil/macrophage RAW 264.7 cell line co-culture by that respective strain alone culture (grown in the same conditions in the absence of neutrophil/macrophage RAW 264.7 cell line). Only the neutrophil/ macrophage RAW 264.7 cell line was kept as a negative control for the assay.

**Growth assay under oxidative stress**

From the mid-log phase cultures of the indicated strains, 0.1% inoculum was inoculated in a 200 µL of fresh LB medium containing methyl viologen (MV) at 250 µM (final conc.). All the growth assays were performed in a sterile 96-well plate (Genaxy) at 37 ⁰C with shaking linearly at 180 CPM (6 mm), and the OD_600_ as a measurement of growth was measured at every 30 min interval for the indicated total time in the Synergy microplate reader (BioTek). Only media without any culture served as a negative control for this assay. The represented data is after background correction.

**ROS quantification**

Bacterial cells were grown at 37 ⁰C with shaking to an OD_600_ of 0.6 (mid-log phase) in LB-medium. Bacterial cells were harvested by centrifugation and washed in sterile 1X PBS. The bacterial cell pellets were resuspended in 1X PBS, and 2’,7’-dichlorofluorescein diacetate (Thermo Fisher Scientific, D399) was added at a final concentration of 100 µM. After incubation for 30 min at 37 ⁰C, the cells were washed with 1X PBS to remove excess dye and transferred to 100 µL/well of a 96-well transparent bottom black well plate (BRAND). MV (250 µM final conc.) was added to the wells containing bacterial cells and incubated at 37 ⁰C with shaking linearly at 180 CPM (6 mm). OD_600_ and fluorescence (excitation/emission at 485/535 nm) were measured every 10 min interval for the indicated total time in the Synergy microplate reader (BioTek). The represented data is after background correction and OD_600_ normalization.

**Quantitative RT-PCR analysis**

Human blood-derived neutrophils were co-incubated with the bacterial strains as described in the above section. After 4 h of incubation, the tissue culture plate was centrifuged at 400g for 5 min to settle down the neutrophils. Sample supernatants containing the bacterial cells were collected and harvested the bacterial cells by centrifugation at maximum speed. After washing the cell pellet with 1X PBS, RNA was extracted from the bacterial cells by the classic phenol-chloroform method. The cDNA synthesis was performed using PrimeScript 1st strand cDNA Synthesis Kit (TakaRa, 28704) according to the manufacturer’s instructions. Amplifications were achieved using a 3-step program on a QuantStudio 5 system (Thermo Fisher Scientific).

**Transformation of pAB3 into WT T6+ cells**

Total plasmids (pAB1, pAB2, and pAB3 present in *A. baumannii* ATCC 17978 strain) were isolated from an overnight culture of *A. baumannii* ATCC 17978 strain grown in LB using plasmid miniprep kit (Thermo Fisher Scientific, K0503). The presence of pAB3 in the plasmid isolate was confirmed by PCR using *tetR1* and *tetR2* primers. The plasmid mixture was transformed into the electro-competent WT T6+ cell (devoid of pAB3) and the transformants were selected on LB agar plate containing sulfamethoxazole/trimethoprim (S&T; 30 µg/mL and 5 µg/mL, respectively). The transformation of pAB3 into WT T6+ competent cells was confirmed by PCR using forward and reverse primers of *tetR1* and *tetR2* genes.

**Generation of knockout strains**

The deletion mutants were created using a homologous-recombination method described previously (2) with some modifications. Briefly, a construct carrying an apramycin cassette (amplified from pMDIAI and having FRT sites on both sides) flanking between 500 bp upstream and 500 bp downstream of the gene of interest was cloned into a pUC18 vector (used as a cloning vector). A PCR product was amplified from the construct using a 125 bp upstream forward primer and a 125 bp downstream reverse primer of the gene of interest listed in Table S5. Around 5 µg of the concentrated PCR gel-purified product was transformed into *A. baumannii* electrocompetent cells harboring pAT02 (which contains Rec_Ab_ system) under IPTG induction (2 mM) and plated on LB-agar containing apramycin (15 µg/mL). The transformants were further passaged on LB-agar containing an increasing concentration of apramycin (15-30 µg/mL). The recombinants were further confirmed by PCR using primers located outside the regions of homology (i.e., 500 bp upstream forward primer and 500 bp downstream reverse primer of the gene of interest) listed in Table S5. Following PCR confirmation and curing of pAT02, a clean knockout (K/O) was created by transforming pAT03 (which contains the FLP recombinase system) under IPTG induction (2 mM). A loss of apramycin resistance confirmed the clean K/O and the losing apramycin-FRT was further confirmed by PCR using apramycin forward and reverse primers listed in Table S5. After curing pAT03, the clean K/O strains were maintained in glycerol (15%) at -80 ⁰C for further use.

**Quantification of intracellular metal content**

A fresh streaked colony of WT T6- and WT T6+ strain on LB-agar plates were used to inoculate in 5 mL minimal medium (M9-medium) supplemented with 1% casamino acids as a nutrient source for O/N at 37 ⁰C with shaking. 0.1% inoculum from the O/N cultures was then subcultured into fresh 100 mL M9-medium (supplemented with 1% casamino acids) supplemented with or without MnCl_2_, ZnSO_4_, or FeCl_3_ at a final concentration of 100 µM and grown at 37 ⁰C with shaking to an OD_600_ of 0.6 (mid-log phase) for each strain. Then MV was added at a final concentration of 100 µM to the culture to induce oxidative stress, and it was grown further at 37 ⁰C with shaking for 4 h. The bacterial cultures (OD_600_ normalized volume) were then transferred to pre-weighed metal-free 50 mL centrifuge tubes and centrifuged to harvest the cell pellet, washed thrice with Milli-Q deionized water, and dried thoroughly. The pellet weight was measured using an analytical balance (G&G). Pellets were digested with 1 mL of 70% HNO_3_ using Milli-Q deionized water as a diluent for O/N at 90 ⁰C and diluted with 9 mL of 3.5% HNO_3_ using Milli-Q deionized water as a diluent. The samples were then subjected to inductively coupled plasma-mass-spectrometry (8900 ICP-MS Triple Quad, Agilent) at the Institute Instrumentation Centre (IIC) in IIT Roorkee. The concentrations were determined by utilizing a standard curve for each metal. Only M9-medium supplemented with 1% casamino acids was used as a control.

**Bacterial killing assay**

*A. baumannii* T6SS- and Δ*mumT* strains were grown in 5 mL of LB-medium containing 100 µM MV and MnCl_2_ at 37 ⁰C with shaking to an OD_600_ of 0.6 (mid-log phase). Cells were harvested from a 2 mL culture, washed with 1X PBS, and dissolved into 50 µL of 1X PBS. Simultaneously, the prey cells were grown in LB medium containing respective selection markers to an OD_600_ of 0.6 (mid-log phase), harvested the cells from 2 mL culture, washed with 1X PBS, and dissolved into 50 µL of 1X PBS. The predator and prey cells were mixed at a ratio of 1:1 and spotted 100 µL mixture on a sterile 0.22 µm syringe filter (Merk Millipore Ltd.) placed on dry LB agar plates. After air-drying inside the hood, the plates were kept at 37 ⁰C for 4 h. The mixed cultures were scraped out and resuspended in 1X PBS. For spot assay, after a serial dilution in 1X PBS, 5 µL from each dilution was spotted onto LB agar containing sodium azide (100 µg/mL) when *E. coli* J53 was used as prey or LB agar containing kanamycin (50 µg/mL) when *E. coli*-pNYL GFP was used as prey. The plates were incubated at 37 ⁰C for O/N and images were taken using a camera. For CFU count, after a serial dilution in 1X PBS, 100 µL from each dilution was spread onto LB agar containing sodium azide (100 µg/mL) when *E. coli* J53 was used as prey, or LB agar containing kanamycin (50 µg/mL) when *E. coli*-pNYL GFP was used as prey, or *P. aeruginosa* agar medium when *P. aeruginosa* was used as prey. The plates were incubated at 37 ⁰C for O/N. The survival percentage of the prey cells was calculated by considering the CFU of prey cells alone as 100%. To measure the prey cells' GFP fluorescence, the predator and prey cells were mixed at a 1:1 ratio in LB-medium in a 96-well transparent bottom black well plate (BRAND). GFP fluorescence was recorded at 485/525 nm to measure prey cells' growth at 37 ⁰C every 3 h.

**Pulse expression studies**

For survival assay, fresh colonies of the indicated strains streaked on LB-agar plates containing kanamycin (50 µg/mL) were used to inoculate in 5 mL of LB-medium containing kanamycin (50 µg/mL) or O/N at 37 ⁰C with shaking. 0.1% inoculum from the O/N cultures was then subcultured into fresh 5 mL of LB-medium with kanamycin (50 µg/mL) containing MnCl_2_ at a final concentration of 250 µM and grown at 37 ⁰C with shaking to an OD_600_ of 0.6 (mid-log phase). MV (250 µM final conc.) was added to the culture and grown for another 2 h. Cells were harvested from a 2 mL culture, washed with 1X PBS, and dissolved into 50 µL of 1X PBS. Simultaneously, *E. coli* J53 as prey cells were grown in LB medium containing sodium azide (100 µg/mL) to an OD_600_ of 0.6 (mid-log phase), harvested the cells from 2 mL culture, washed with 1X PBS, and dissolved into 50 µL of 1X PBS. The predator and prey cells were mixed at a ratio of 1:1, arabinose (0.2% w/v final concentration) was added for AbsR28 expression and spotted the 100 µL mixture on a sterile 0.22 µm syringe filter (Merk Millipore Ltd.), placed on dry LB agar plates. After air-drying inside the hood, the plates were kept at 37 ⁰C for O/N. The mixed cultures were scraped out and resuspended in 1X PBS. After a serial dilution in 1X PBS, 100 µL from each dilution was spread onto LB agar containing sodium azide (100 µg/mL). The plates were incubated at 37 ⁰C for O/N. The survival percentage of the prey cells was calculated by considering the CFU of prey cells alone as 100%. To check gene expression by qRT-PCR, the cells were grown in LB containing MnCl_2_ at a final concentration of 250 µM and grown at 37 ⁰C with shaking to an OD_600_ of 0.6 (mid-log phase). MV (250 µM final conc.) and arabinose (0.2% w/v final concentration) were added to the media and incubated for 4 h. RNA was extracted, and qRT-PCR was performed as described above. For the Hcp-Western blot, the cells were grown in LB containing MnCl_2_ at a final concentration of 250 µM and grown at 37 ⁰C with shaking to an OD_600_ of 0.6 (mid-log phase). MV (250 µM final conc.) and arabinose (0.2% w/v final concentration) were added to the media and incubated for a further 4 h. Cell lysate (CL) and cell-free supernatant (S) were run on a SDS-PAGE and performed Western blot.

**Isothermal calorimetry**

ITC titrations were performed using MicroCal PEAQ-ITC (Malvern Panalytical). After degassing, 600 µM MnCl_2_ was placed into the syringe and 30 µM *in vitro* transcribed AbsR28 was placed in the reaction cell. ITC was performed over 20 injections, each 1.8 µl of MnCl_2_ with an interval of 120 s to allow for equilibration of the mixture between injections at a constant stirring of 500 rpm. All reactions were performed in a buffer containing 10 mM Tris, 100 mM KCl, and pH 8.0 at 25 ⁰C. A control experiment was performed at the same condition in the absence of sRNA. Analysis after subtracting the control data was performed using the Origin version 7.0 software provided with the system, and data was fitted as an independent binding model.

***In vitro* RNA transcription and 5'-end labeling**

T7 transcription was performed using the T7 RNA polymerase (Thermo Scientific, EP0111) according to the manufacturer’s instructions. The DNA contamination was removed by incubating the transcript RNA with DNase I (Thermo Scientific, EN0521) according to the manufacturer’s instructions. Complete transcripts were obtained by phenol-chloroform extraction, running on a long Urea-PAGE followed by gel purification and concentrated by sodium acetate precipitation. To perform 5'-end labeling, transcripts were dephosphorylated with FastAP (Thermo Scientific, EF0651) and 5'-labeled with [^32^P]-γ-ATP using T4 polynucleotide kinase (Thermo Scientific, EK0032) with forward reaction buffer according to the manufacturer’s protocol. Radiolabeled transcripts were purified by running a long Urea-PAGE followed by gel purification and concentrated by sodium acetate precipitation.

***In vitro* structural probing**

*In vitro* transcribed 5'-labeled AbsR28 (2.5 pmol) was incubated in a modified 2X in-line buffer (100 mM Tris-HCl pH 7.0 and 1 M KCl) at a range of MnCl_2_ concentrations containing yeast RNA (1 µg/reaction) for 40 h at room temperature. Afterward, 2 µL of 25 mM lead(II) acetate (Sigma-Aldrich, 215902) stock was added to each of the 10 µL reactions and incubated for precisely 2 min at 37 ⁰C. RNaseT1 ladder was generated by incubating 5'-labeled AbsR28 with RNaseT1 (0.1 U/µL and 1.0 U/µL) in 1X sequencing buffer (Ambion, AM2283) for 3 min at 55°C. Alkaline RNA ladders were generated by incubating 5'-labeled AbsR28 in 1X alkaline buffer (Ambion, AM2283) for 5 min at 90 ⁰C. All reactions were stopped immediately by adding a stop buffer (Ambion, AM2283). After phenol:chloroform:isoamyl alcohol puriﬁcation, RNA pellets were dissolved in loading buffer II (Ambion, AM2283). All samples were denatured for 3 min at 95 ⁰C and loaded on 10% PAGE/7 M urea sequencing gels at a constant 15 Watt. After gel drying for 2 h at 80 ⁰C, bands were visualized using a phosphoimager (Typhoon FLA 7000, GE Healthcare) and ImageQuant software.

**Gel retardation assay**

Unlabeled *tssM* *in vitro* transcripts (250 nt upstream and 250 nt downstream from ATG) at a fixed concentration of 20 pmol and full-length AbsR28 *in vitro* transcripts at an increasing concentration were used for the gel retardation assay. The 10X structure buffer (100 mM Tris-HCl pH 7.0 and 1 M KCl) was added at a final concentration of 1X to the mRNA and the mRNA was allowed to re-nature at 37 ⁰C for 15 min. Yeast RNA (Ambion, AM2283) was added to the reaction mix at a final concentration of 1 µg/reaction and AbsR28 was added to the tubes at an increasing concentration. MnCl_2_ was added to the reaction mix at a final concentration of 10 mM. After incubation at 37 ⁰C for 60 min, 6X RNA native loading buffer (Ambion) was added to stop the reaction and resolved on a 6% native PAGE at 4 ⁰C in 0.5% TBE at a constant current of 40 mA for 6 h. The gel was stained with SYBR Safe, and visualized the RNA bands using a phosphorimager (Typhoon FLA 9000, GE Healthcare) and ImageQuant software.

**RNase E-mediated degradation assay**

The *in vitro* transcript *tssM* (250 nt upstream and 250 nt downstream from ATG) was 5'-labeled with [^32^P]-γ-ATP as described above. 5'-labeled *tssM* mRNA (5 pmol) was denatured at 65 ⁰C for 2 min and chilled on ice for 5 min. The 10X structure buffer (100 mM Tris-HCl pH 7.0 and 1 M KCl) was added at a final concentration of 1X to the mRNA and the mRNA was allowed to re-nature at 37 ⁰C for 15 min. Yeast RNA (Ambion, AM2283) was added to the reaction mix at a final concentration of 1 µg/reaction. Unlabeled AbsR28 (35 pmol) and purified *A. baumannii* Hfq72 or *E. coli* Hfq protein (5-fold molar excess in hexamer over *tssM* transcripts) were added to the tubes. MnCl_2_ was added to the reaction mix at a final concentration of 10 mM. The reaction mixture was incubated at 37 ⁰C for 60 min. To initiate the RNase E-mediated degradation, purified RNase E (only the catalytic amino-terminal domain) at 10-fold molar excess over *tssM* transcripts was added to the reaction mixture and incubated further at 37 ⁰C for 210 min (0 min denotes the initial time point when RNase E was added). EDTA (2.5 µL from 50 mM stock) and Proteinase K (2.5 µL from 20 mg/mL stock) were added to each reaction mixture and incubated at 50 ⁰C for 10 min. Samples were purified immediately using a 2X precipitation buffer supplied with the RNase T1 kit (Ambion, AM2283) according to the manufacturer’s instructions. RNA pellets were dissolved in loading buffer II (Ambion, AM2283) and denatured for 3 min at 95° C. RNA cleavage products were resolved on 6% native PAGE at a constant 15 Watt. After gel drying for 2 h at 80 ⁰C, bands were visualized using a phosphoimager (Typhoon FLA 9000, GE Healthcare) and ImageQuant software.

**Mice infection model for *A. baumannii* pneumonia**

All animal experiments under protocol BT/IAEC/2018/07 were reviewed and approved by the Institute Animal Ethics Committee of the Indian Institute of Technology Roorkee. The mice were anesthetized and infected intranasally with 20 µL of inoculum containing 4×10^4^ CFU of the indicated strains. Mice were euthanized at 36 h of infection, and the lungs and livers were harvested, immediately transferred on ice, and washed with ice-cold 1X sterile PBS. The harvested organs were chopped into pieces to enumerate the bacterial burden, histopathology, and Western blot analysis.

**REFERENCES**

1. Weber BS, Miyata ST, Iwashkiw JA, Mortensen BL, Skaar EP, Pukatzki S, Feldman MF. 2013. Genomic and functional analysis of the type VI secretion system in *Acinetobacter*. PLoS One 8:e55142.
2. Tucker AT, Nowicki EM, Boll JM, Knauf GA, Burdis NC, Trent MS, Davies BW. 2014. Defining gene-phenotype relationships in *Acinetobacter baumannii* through one-step chromosomal gene inactivation. mBio 5:e01313–14.

**SUPPLEMENTAL FIGURE AND LEGENDS**

**
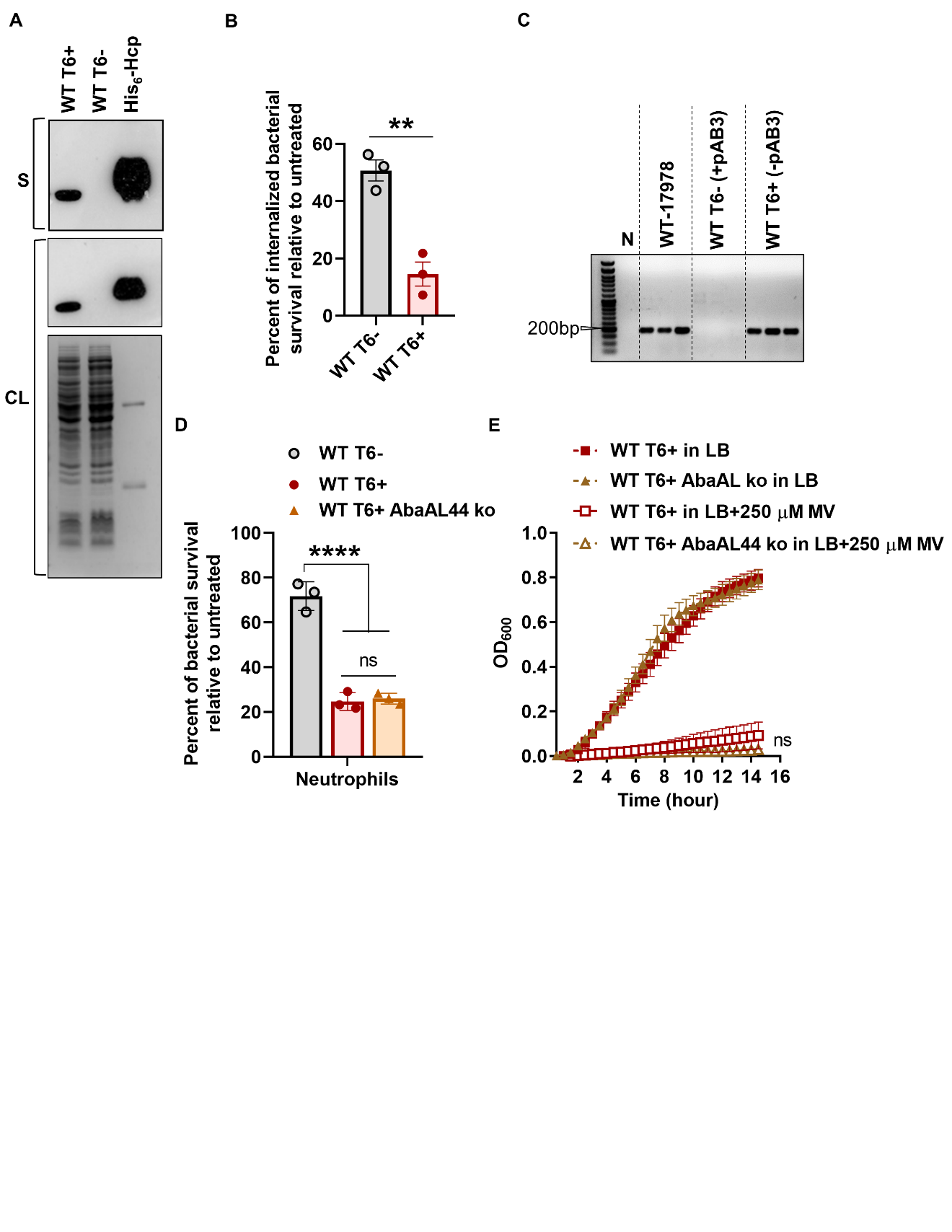
**

**FIG S1** *A. baumannii* T6+ cells are sensitive to oxidative stress due to inadequate uptake of Mn^2+^. (**A**) The cell-free supernatants (S) of the indicated strains were collected, and the Hcp-secretion profile of the strains was checked by Western blot (upper panel). The Hcp-expression in cell pellet of WT T6- and WT T6+ strains was assessed (lower panel) and gel image below is provided as a loading control. Purified His_6_-Hcp was used as a positive control for Western Blot. (**B**) Human blood-derived neutrophils were co-incubated with either *A. baumannii* ATCC 17978 wild-type T6SS- (WT T6-) or wild-type T6SS+ (WT T6+) strain for 4 h at the MOI of 1. The cells were washed after incubation with gentamycin (300 µg/mL) for 2 h. Neutrophils were lysed using 0.04% Triton X-100. Cell lysates were serially diluted and plated onto Leeds *Acinetobacter* medium plates. The percentage of bacterial survival was enumerated by accounting for the respective untreated control (without phagocytic cells) as 100%. The data represents the mean of biological triplicates ± SEM. Statistical significance was determined using Student’s t-test. ** denotes p-value <0.01. (**C**) The presence and absence of *clsc2* (which is present in AbaAL44 island) were checked by PCR in individual colonies of each strain used in the study. Amplification and no amplification for *clsC2* indicate the presence and absence of AbaAL44, respectively. The PCR amplification for *clsC2* was observed in WT T6+ strain whereas no amplification was observed in WT T6- strain. Data representation has been provided for three colonies of each strain. (**D**) The percentage of bacterial survival was enumerated after incubation with human blood-derived neutrophils for 4 h at the MOI of 1 by accounting for the respective untreated control. (**E**) Growth of the WT T6+ with AbaAL44 island (WT T6+) and WT T6+ AbaAL44 deletion mutant (WT T6+ AbaAL44 ko) was assessed in LB supplemented with or without MV. Statistical significance was determined using the Student’s t-test (B), one-way ANOVA test with Tukey’s multiple comparisons (D). ** denotes p-value <0.01, **** denotes p-value <0.0001, ns denotes not significant.


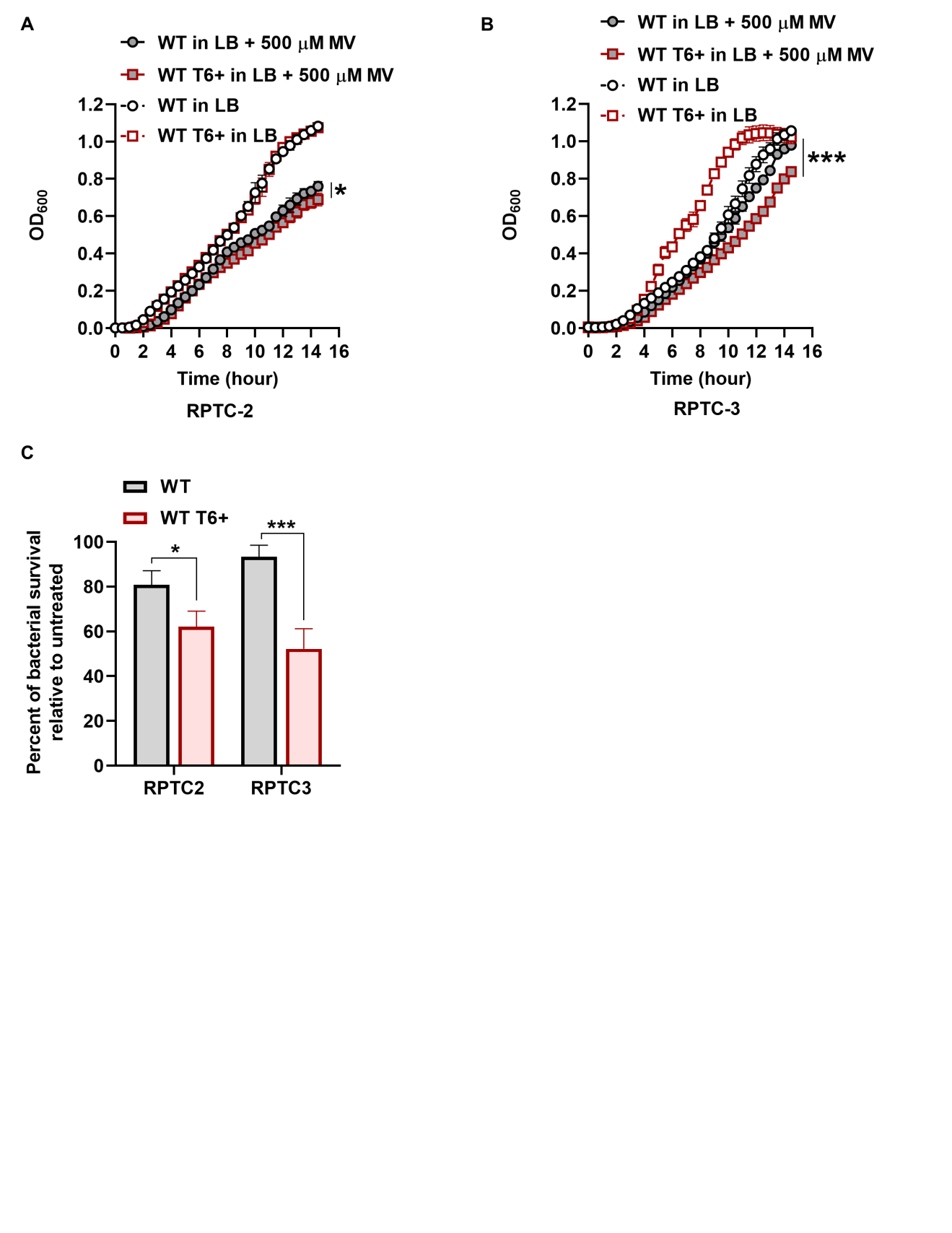


**FIG S2** The T6+ cells of clinical isolates are more susceptible to oxidative stress. (**A and B**). The T6+ strains of the clinical isolates were obtained using the same procedure as described for ATCC 17978 and compared their growth with their respective wild-type (WT) strain in LB medium supplemented with MV. A higher concentration of MV was used because concentrations up to 250 µM did not show any growth difference compared to LB medium alone for these isolates. The data represents mean ± SD. Statistical significance was determined using the Student’s t-test. (**C**) Human blood-derived neutrophils were co-incubated with the indicated strains for 4 h at an MOI of 1. The percentage of bacterial survival was enumerated by accounting for the respective untreated control (without phagocytic cells) as 100%. The data represents the mean of biological triplicates ± SEM. Statistical significance was determined using the multiple comparison two-way ANOVA test with the Sidak correction for multiple comparisons, comparing the means of each group to one another. * denotes p-value <0.05, **** denotes p-value <0.0001.


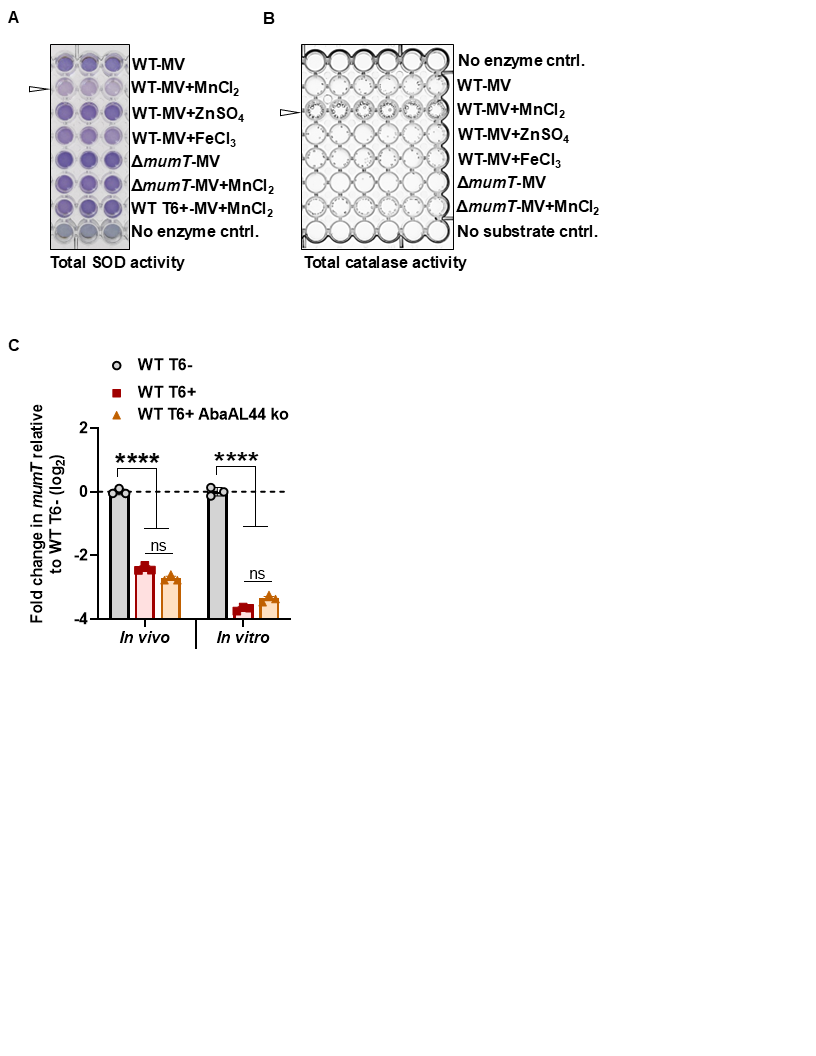


**FIG S3** *A. baumannii* T6+ cells are sensitive to oxidative stress due to inadequate uptake of Mn^2+^, which is required for SOD and catalase activity. (**A**) Total SOD activity was determined using nitroblue tetrazolium (NBT) as a substrate. An equal amount of bacterial cell lysates (grown in M9-media containing casamino acid supplemented with MV + metal ions) were used in this assay. Active SOD causes an inhibition of the photochemical reduction of NBT in the presence of riboflavin which can be visualized by the naked eye. The highest SOD-mediated inhibition was observed in the presence of MnCl_2_ (denoted by an empty triangle). (**B**) Total catalase activity was determined using H_2_O_2_ as a substrate. An equal amount of bacterial cell lysates (grown in M9-media containing casamino acid supplemented with MV + metal ions) were used in this assay. Active catalase breaks down H_2_O_2_ to H_2_O and O_2_, which produces air bubbles that can be visualized by the naked eye. (**C**) The expression of *mumT* was determined in the indicated strains in both *in vivo* (co-incubated with neutrophils) and *in vitro* (in the presence of MV) by qRT-PCR. Statistical significance was determined using one-way ANOVA test with Tukey’s multiple comparisons. **** denotes p-value <0.0001.

**
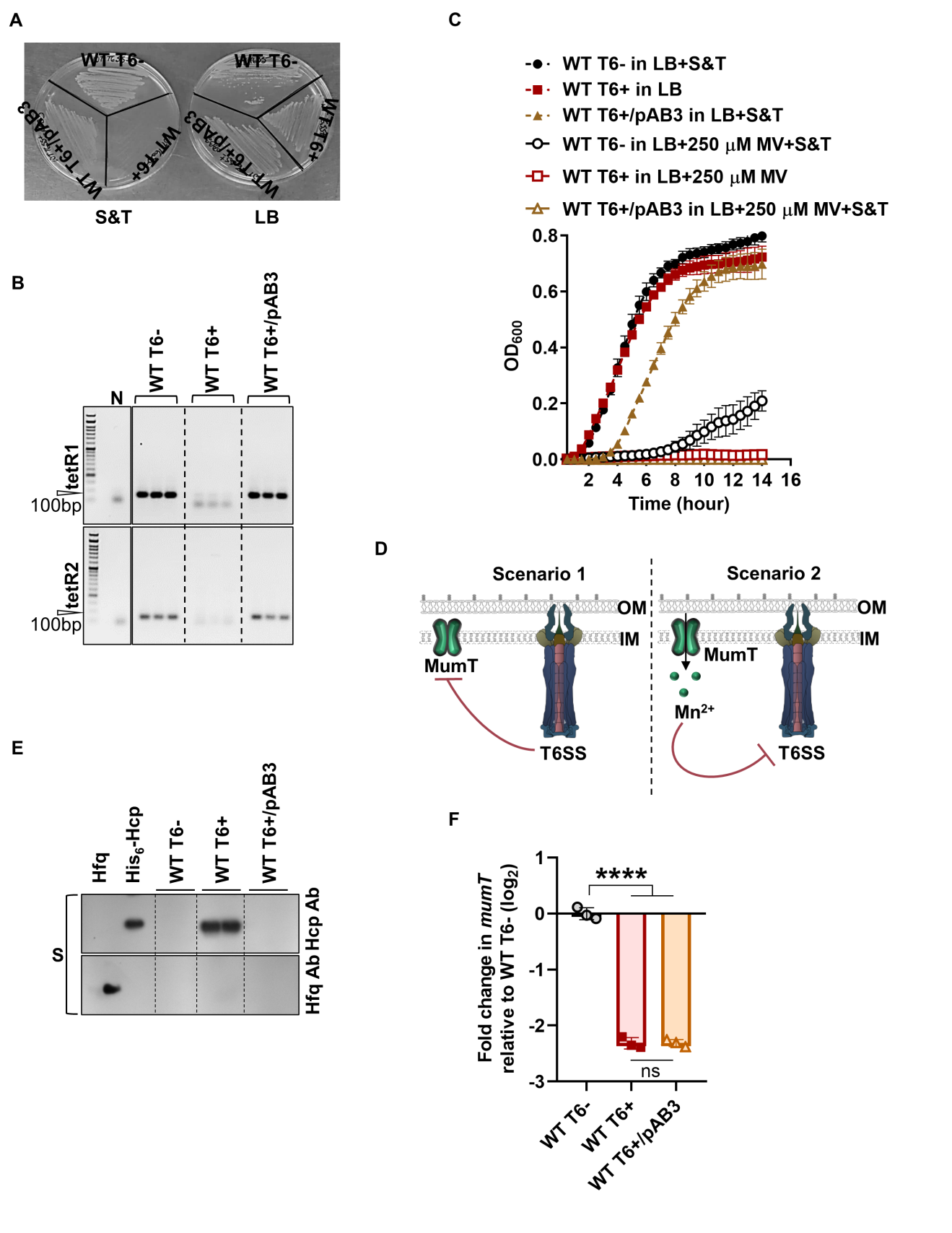
**

**FIG S4** pAB3 has no role in mitigating oxidative stress. (**A**) pAB3 was transformed into the WT T6+ competent cells, and the transformants were confirmed by streaking on LB agar plate containing sulfamethoxazole/trimethoprim (S&T; 30 µg/mL and 5 µg/mL, respectively), where the cells lacking pAB3 will not grow. (**B**) The transformation of pAB3 into WT T6+ competent cells was confirmed by PCR using forward and reverse primers of *tetR1* and *tetR2,* which are present in pAB3. Bacterial cells devoid of pAB3 did not show any amplification. (**C**) Growth of WT T6-, WT T6+, and WT T6+/pAB3 cells in LB supplemented with MV (at a final concentration of 250 µM). Following the assay, the presence of pAB3 was confirmed in both WT T6- and WT T6+/pAB3 cells through PCR analysis. The data represents four biological replicates in technical duplicate with a standard deviation (SD) of the mean. (**D**) Schematic representation of two possible scenarios. (**E**) The cell-free supernatants (S) of the indicated strains were collected, and the Hcp-secretion profile of the strains (in duplicates) was checked by Western blot. Hfq antibody was used to confirm that the supernatants were free from the bacterial cell. (**F**) The transcripts level of *mumT* in WT T6-, WT T6+, and WT T6+/pAB3 strains in LB supplemented with MV was determined by qRT-PCR. The data represents the mean ± SD. Statistical significance was determined using the Tukey's multiple comparisons. **** denotes p-value <0.0001, ns denotes not significant.

**
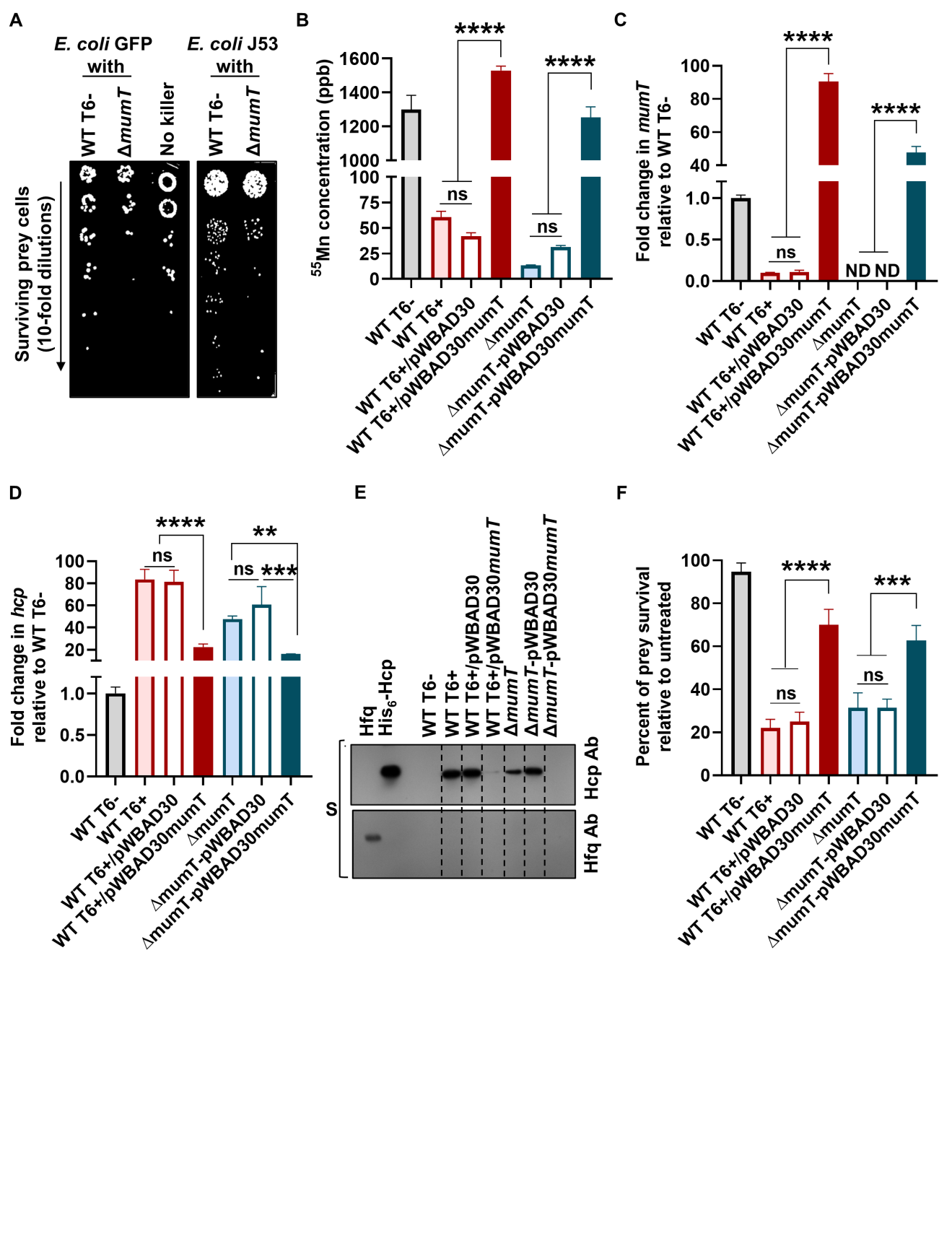
**

**FIG S5** Complementation of *mumT* results in a decrease in T6SS expression in *A. baumannii* under oxidative stress. (**A**) Recovery of surviving prey cells after co-incubation with WT T6- or Δ*mumT*. (**B**) Intracellular ^55^Mn was quantified by ICP-MS. The bacterial strains were grown in minimal media (M9-media) containing casamino acid as a nutrient source supplemented with MV + MnCl_2_ (at a final concentration of 100 µM), and the intracellular Mn^2+^ concentration was measured in cell pellets by ICP-MS. The data represents three biological replicates with a standard deviation (SD) of the mean. (**C and D**) The bacterial strains were grown in LB supplemented with MnCl_2_ (250 µM) till the mid-log phase (OD_600_~0.6) and incubated further with MV (250 µM). RNA was extracted from the cells after arabinose induction, and cDNA was prepared. The expression level of *hcp* and *mumT* transcripts in the strains was determined by qRT-PCR. The data represents the mean ± SD. (**E**) The cell-free supernatants (S) from the above mentioned experiment were collected, and the Hcp-secretion profile of the strains was checked by Western blot. Hfq antibody was used to confirm that the supernatants were free from the bacterial cell. (**F**) In the T6SS competition assay, prey cells (*E.coli* J53) were subjected to killing by incubation with the indicated strains. The survival percentage of prey cells was enumerated by accounting for the respective untreated control (without predator/killer cells) as 100%. The data represents the mean of three biological replicates ± SD. For all the assays, WT T6- and WT T6+ cells were used as T6- and T6+ control, respectively. Statistical significance was determined using the one-way ANOVA test with Tukey's multiple comparisons. ** denotes p-value <0.01, *** denotes p-value <0.001, **** denotes p-value <0.0001, ns denotes not significant. ND denotes not detected.

**
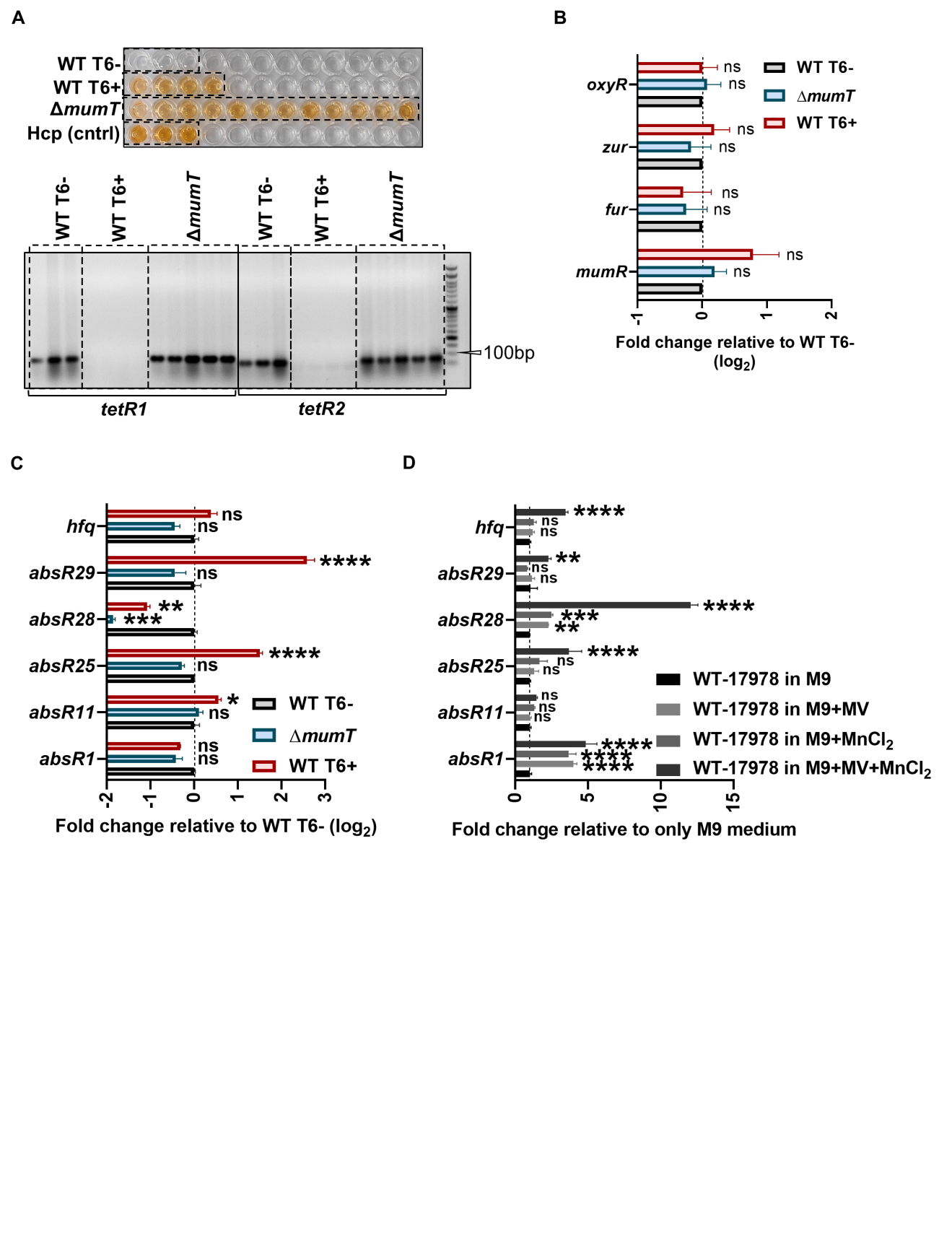
**

**FIG S6** Supplementation of MnCl_2_ results in an increase of AbsR28 at the transcript level. (**A**) Hcp-ELISA assay was performed for Δ*mumT* strain (upper panel) and wells that exhibited Hcp-secretion positive were picked up to determine the presence of pAB3 by PCR using forward and reverse primers of *tetR1* and *tetR2,* which are present in pAB3. Bacterial cells devoid of pAB3 did not show any amplification. WT T6- and WT T6+ strains were used as pAB3 positive and pAB3 negative control for this assay, respectively. Gel image of five samples is represented. (**B**) The expression of several transcriptional regulators of T6SS was determined by qRT-PCR, where the bacterial strains were grown in an LB medium. The data represents the mean ± SD. (**C**) The expression of several sRNAs and *hfq* was determined by qRT-PCR, where the bacterial strains were grown in an LB medium. The data represents the mean ± SD. (**D**) The expression of several sRNAs and *hfq* was determined by qRT-PCR, where the bacterial strains were grown in M9-media containing casamino acid supplemented with either MV or MnCl_2_ alone or MV+MnCl_2_ (250 µM). Statistical significance was determined using the multiple comparison two-way ANOVA test with the Sidak correction for multiple comparisons, comparing the means of each group to one another. * denotes p-value <0.05, ** denotes p-value <0.01, *** denotes p-value <0.001, **** denotes p-value <0.0001, ns denotes not significant.

**
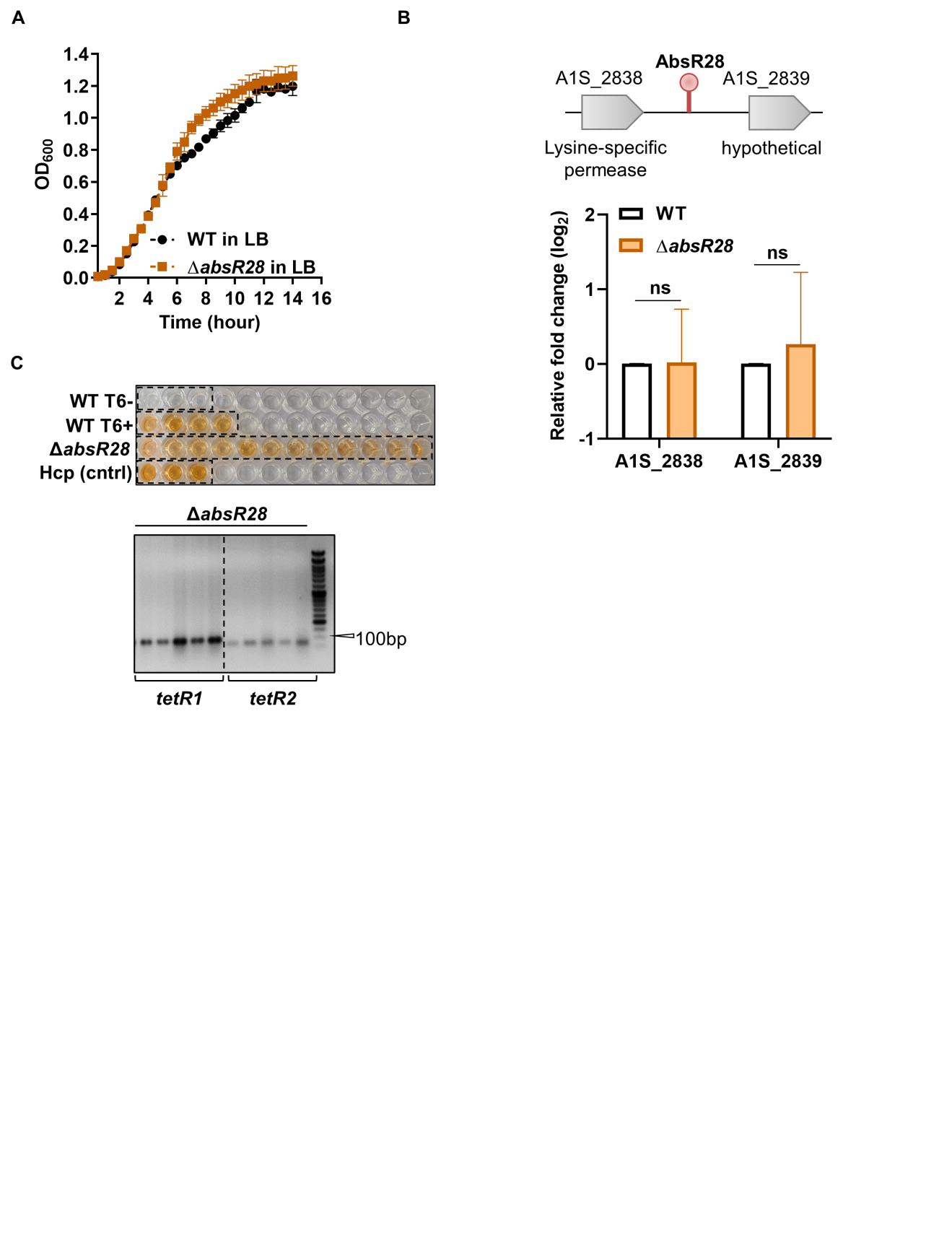
**

**FIG S7** ∆*absR28* cells are predominant in T6+ despite losing pAB3. (**A**) The growth of WT T6- and ∆*absR28* cells in LB medium was assessed at the indicated time points. (**B**) Genomic location of AbsR28 in *A. baumannii* ATCC 17978 (NCBI Ref. seq. CP000521.1) (upper panel). WT and Δ*absR28* strains were grown in LB supplemented with MnCl_2_ (250 µM) till the mid-log phase (OD_600_~0.6) and incubated further with MV (250 µM) for 2 h. RNA was extracted from the cells, and cDNA was prepared. The expression of A1S_2828 and A1S_2839 transcripts, which are immediate upstream and downstream of AbsR28, respectively, was checked by qRT-PCR. No change in the expression in Δ*absR28* for both the genes with respect to WT confirms no polar effect of Δ*absR28*. The data represents the mean ± SD. Statistical significance was determined using the multiple comparison two-way ANOVA test with the Sidak correction for multiple comparisons, comparing the means of each group to one another. ns denotes not significant. . (**C**) Hcp-ELISA assay was performed for Δ*absR28* strain (upper panel) and wells that exhibited Hcp-secretion positive were picked up to determine the presence of pAB3 by PCR using forward and reverse primers of *tetR1* and *tetR2,* which are present in pAB3. Bacterial cells devoid of pAB3 did not show any amplification. Gel image of five samples is represented.

**
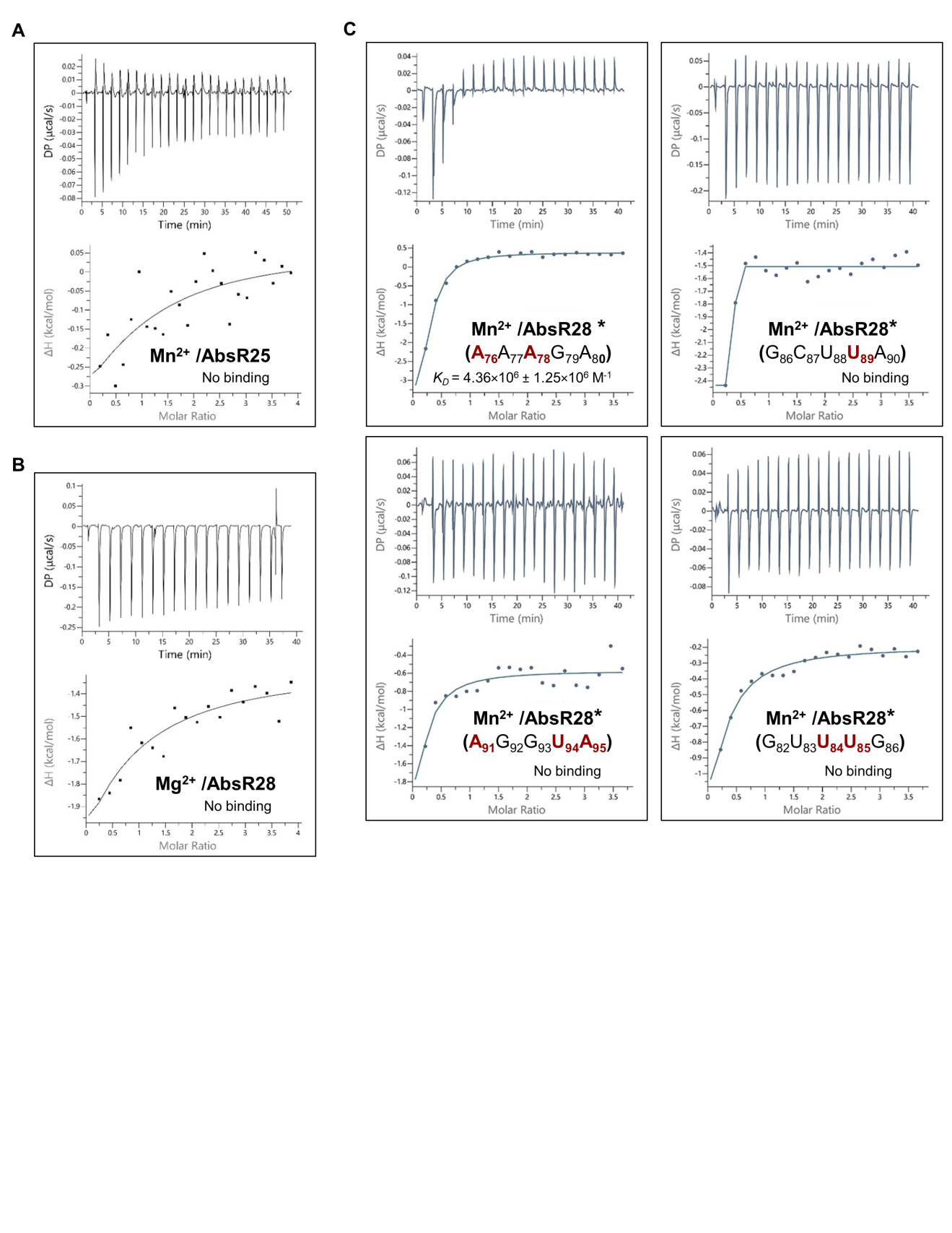
**

**FIG S8** Mn^2+^ binds to AbsR28. (**A**) Isothermal titration calorimetry (ITC) of Mn^2+^ to AbsR25 as nonspecific sRNA control. (**B**) ITC of Mg^2+^ to AbsR28 as nonspecific metal ion control. (**C**) ITC of Mn^2+^ to AbsR28 mutants (*). Mutated nucleotides are highlighted.

**
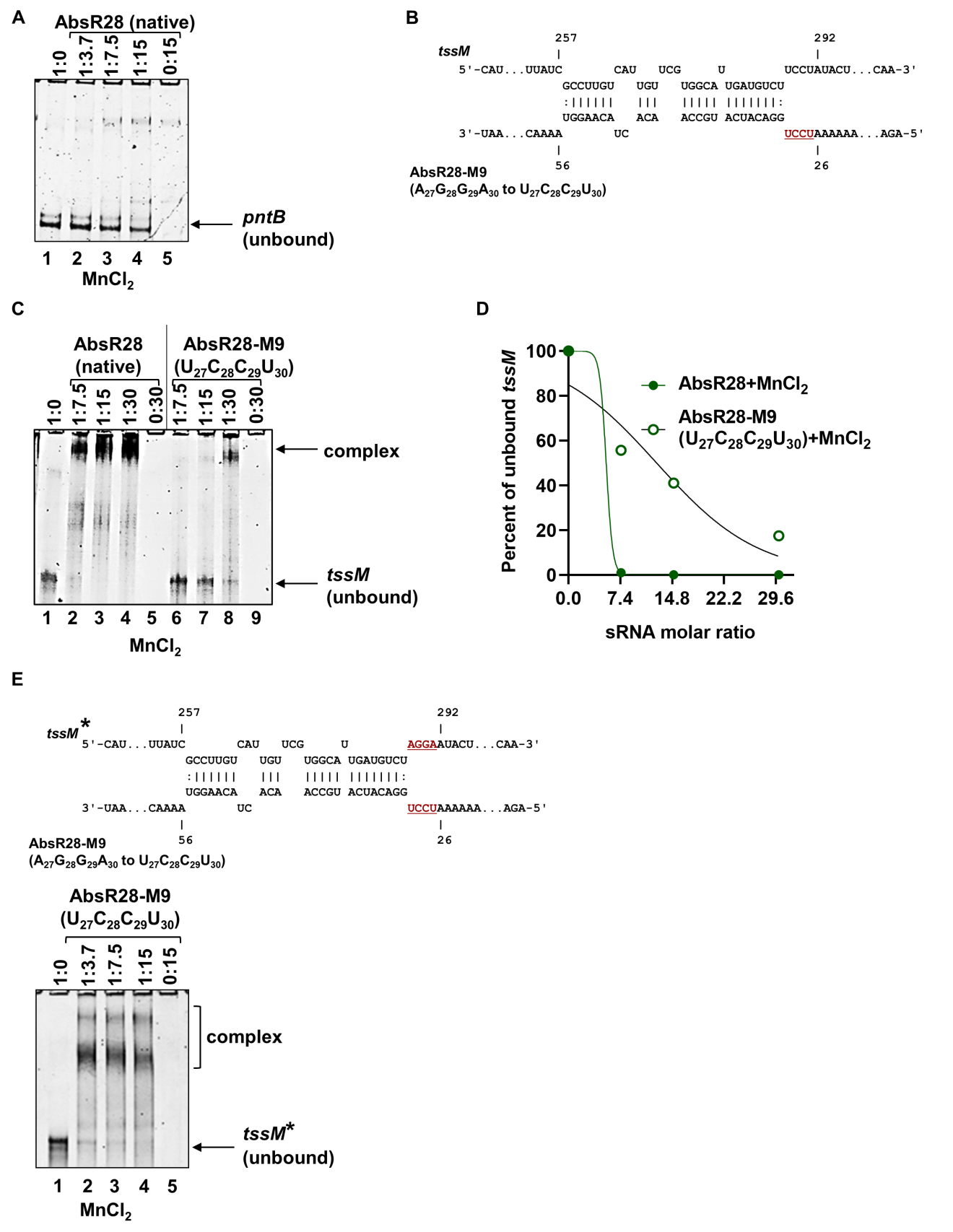
**

**FIG S9** AbsR28 base pairs with *tssM* mRNA in the presence of Mn^2+^. (**A**) Gel retardation assay of unlabeled *pntB* *in vitro* transcripts (used as a negative mRNA control) with unlabeled full-length AbsR28 *in vitro* transcripts in structure buffer containing MnCl_2_. (**B**) The position of the four nucleotides of the seed region of AbsR28 is mutated (AbsR28-M9) and shown in red underlined. (**C**) Gel retardation assay of unlabeled *tssM* *in vitro* transcripts with unlabeled full-length AbsR28 or AbsR28-M9 *in vitro* transcripts in structure buffer containing MnCl_2_. (**D**) Quantification of unbound *tssM* obtained from Figure S8C (n = 2 independent experiments) is shown using ImageJ software. Nonlinear regression was used to fit the curve. (**E**) Gel retardation assay of unlabeled compensatory mutation in *tssM* mRNA (*tssM**) *in vitro* transcripts with unlabeled AbsR28-M9 *in vitro* transcripts in structure buffer containing MnCl_2_.

**
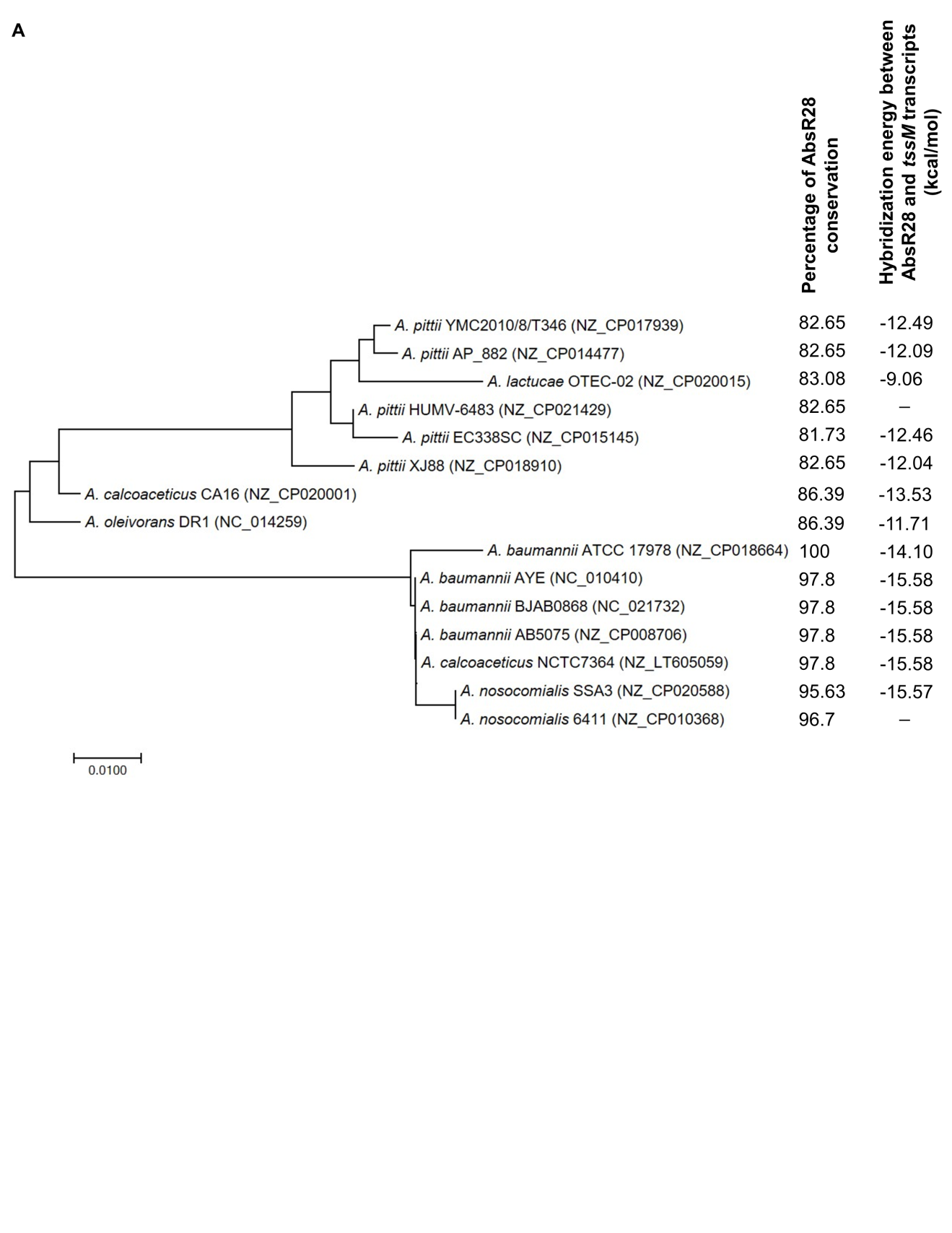
**

**FIG S10** The base pairing of AbsR28 and *tssM* transcripts are mostly conserved amongst *Acinetobacter* species. Conservation analysis of the AbsR28 coding sequence was conducted amongst *Acinetobacter* species using GLASSgo. CopraRNA predictions were used to evaluate potential base pairing between AbsR28 and *tssM* transcripts (denoted as hybridization energy at kcal/mol). Sequences spanning 200 nt upstream and 100 nt downstream of the *tssM* AUG were utilized to investigate possible base pairing regions with AbsR28. *Acinetobacter* species that did not exhibit any interaction within the top 200 targets are denoted by a "-."

**Table S1.** CopraRNA search results for each of the 31 sRNAs (whose sequences were bioinformatically predicted) that showed T6SS genes as hit.

| **small RNA** | **Component of T6SS** | **Binding energy (kcal/mol)** |
| --- | --- | --- |
| AbsR1 | type VI secretion system ATPase TssH | -7.49 |
| AbsR2 | Nil | NA |
| AbsR3 | Nil | NA |
| AbsR4 | type VI secretion system baseplate subunit TssG | -9.47 |
| AbsR5 | type VI secretion system protein TssA | -15.12 |
| AbsR6 | Nil | NA |
| AbsR7 | Nil | NA |
| AbsR8 | type VI secretion system protein TssA | -8.33 |
|  | type VI secretion system baseplate subunit TssG | -7.58 |
| AbsR9 | Nil | NA |
| AbsR10 | Nil | NA |
| AbsR11 | type VI secretion system-associated protein TagF | -14.58 |
| AbsR12 | type VI secretion system baseplate subunit TssK | -11.06 |
|  | type VI secretion system-associated protein TagF | -8.92 |
|  | type VI secretion system tube protein Hcp | -8.72 |
|  | type VI secretion system baseplate subunit TssG | -8.71 |
| AbsR13 | type VI secretion system baseplate subunit TssK | -15.64 |
| AbsR14 | type VI secretion system contractile sheath large subunit TssC | -22.86 |
| AbsR15 | type VI secretion protein TssM | -14.76 |
| AbsR16 | Nil | NA |
| AbsR17 | type VI secretion system-associated protein TagF | -12.23 |
| AbsR18 | type VI secretion protein TssM | -9.37 |
| AbsR19 | Nil | NA |
| AbsR20 | type VI secretion system protein | -13.31 |
| AbsR21 | type VI secretion system baseplate subunit TssG | -14.7 |
|  | type VI secretion system protein | -10.76 |
| AbsR22 | Nil | NA |
| AbsR23 | Nil | NA |
| AbsR24 | Nil | NA |
| AbsR25 | type VI secretion system baseplate subunit TssK | -14.15 |
|  | type VI secretion system protein TssA | -13.57 |
| AbsR26 | type VI secretion system baseplate subunit TssK | -9.44 |
| AbsR27 | Nil | NA |
| AbsR28 | type VI secretion protein TssM | -18.29 |
|  | type VI secretion system baseplate subunit TssG | -10.54 |
| AbsR29 | type VI secretion system protein | -14.9 |
|  | type VI secretion system-associated protein TagF | -12.73 |
| AbsR30 | Nil | NA |
| AbsR31 | type VI secretion system baseplate subunit TssK | -15.64 |

**Table S2.** Genes with altered expression (log_2_<-1 and >+1) in Δ*absR28* strain relative to WT T6- grown in LB medium supplemented with MV+MnCl_2_, both at final conc. of 250 µM (represented in Figure 3B).

| **Gene locus** | **Annotation (KEGG) and function** | **Fold change (log_2_)** |
| --- | --- | --- |
| A1S_2668 | Phosphoenolpyruvate carboxykinase (pyruvate metabolism) | -1.145 |
| A1S_0804 | Trehalose-6-phosphate phosphatase (Starch and sucrose metabolism) | 1.718 |
| A1S_0140 | NAD-linked malate dehydrogenase (pyruvate metabolism) | 1.583 |
| A1S_1341 | Enoyl-CoA hydratase/carnithine racemase (fatty acid degradation) | 1.026 |
| A1S_3362 | Hypothetical protein (glycerophospholipid metabolism) | 2.034 |
| A1S_0041 | Putative linoleoyl-CoA desaturase (biosynthesis of unsaturated fatty acids) | 1.279 |
| A1S_3356 | Putative flavoprotein monooxygenase (purine metabolism) | -1.326 |
| A1S_2686 | Carbamoyl-phosphate synthase small chain (pyrimidine metabolism) | -1.033 |
| A1S_1528 | Hypothetical protein (amino acid metabolism) | -1.152 |
| A1S_0971 | Methionine synthase (cysteine and methionine metabolism) | -3.113 |
| A1S_0737 | 5-Methyltetrahydropteroyltriglutamate-homocysteine methyltransferase (cysteine and methionine metabolism) | -1.213 |
| A1S_1376 | Acyl-CoA dehydrogenase (amino acid degradation) | -1.238 |
| A1S_1341 | Enoyl-CoA hydratase/carnithine racemase (amino acid degradation) | 1.026 |
| A1S_1375 | Putative propionyl-CoA carboxylase (β subunit) (amino acid degradation) | -1.082 |
| A1S_1277 | Allophanate hydrolase subunit 2 (arginine biosynthesis) | -1.312 |
| A1S_1283 | Putative amidase (arginine biosynthesis) | -1.543 |
| A1S_1528 | Hypothetical protein (arginine and proline metabolism) | -1.152 |
| A1S_3415 | Maleylacetoacetate isomerase (tyrosine metabolism) | 1.121 |
| A1S_3414 | Fumarylacetoacetase (tyrosine metabolism) | 1.015 |
| A1S_1336 | Hypothetical protein (phenylalanine metabolism) | 1.758 |
| A1S_1337 | Phenylacetic acid degradation B (phenylalanine metabolism) | 1.61 |
| A1S_1338 | Hypothetical protein (phenylalanine metabolism) | 1.832 |
| A1S_1340 | Phenylacetate-CoA oxygenase/reductase PaaK subunit (phenylalanine metabolism) | 1.26 |
| A1S_1335 | Phenylacetic acid degradation protein paaN (phenylalanine metabolism) | 1.138 |
| A1S_1341 | Enoyl-CoA hydratase/carnithine racemase (phenylalanine metabolism) | 1.026 |
| A1S_1075 | D-amino-acid dehydrogenase (phenylalanine metabolism) | 1.268 |
| A1S_1341 | Enoyl-CoA hydratase/carnithine racemase (phenylalanine metabolism) | 1.026 |
| A1S_2448 | Putative phosphate transporter (two-component system) | 1.628 |
| A1S_2301 | Amino acid ABC transporter permease protein (ABC transporter) | -1.425 |
| A1S_0466 | Sec-independent protein translocase protein (twin-arginine translocation system) | 1.205 |
| A1S_2448 | Putative phosphate transporter (two-component system) | 1.628 |
| A1S_0140 | NAD-linked malate dehydrogenase (two-component system) | 1.583 |
| A1S_1978 | Response regulator protein (two-component system) | -1.021 |
| A1S_2196 | Membrane-associated dicarboxylate transport protein (two-component system) | 1.344 |
| A1S_1925 | Cytochrome d terminal oxidase polypeptide subunit II (two-component system) | -1.073 |
| A1S_1384 | CinA-like protein (nicotinate and nicotinamide metabolism) | 1.627 |
| A1S_0566 | Pyridine nucleotide transhydrogenase (proton pump) α subunit (part1) (nicotinate and nicotinamide metabolism) | -2.918 |
| A1S_0567 | Pyridine nucleotide transhydrogenase (proton pump) α subunit (part2) (nicotinate and nicotinamide metabolism) | -3.028 |
| A1S_0568 | Pyridine nucleotide transhydrogenase β subunit (nicotinate and nicotinamide metabolism) | -2.95 |
| A1S_0466 | Sec-independent protein translocase protein (protein export) | 1.205 |
| A1S_1221 | Hypothetical protein (mismatch repair) | 1.118 |

**Table S3.** Bacterial strains were used in this study.

| **Strains** | **Name used in this study** | **Description** | **Reference** |
| --- | --- | --- | --- |
| *Acinetobacter baumannii* ATCC 17978 | WT | Wild-type (WT) strain, AbaAL44+ | ATCC, USA |
| *Acinetobacter baumannii* ATCC 17978 T6- | WT T6- | T6SS repressed, pAB3 present, AbaAL44-, S/T^R^ | This study |
| *Acinetobacter baumannii* ATCC 17978 T6+ | WT T6+ | T6SS expressed, pAB3 absent, AbaAL44+ | This study |
| *Acinetobacter baumannii* ATCC 17978 T6+ AbaAL44 ko | WT T6+ AbaAL44 ko | T6SS expressed, pAB3 absent, AbaAL44- | This study |
| *Acinetobacter baumannii* ATCC 17978 T6+/ pWBAD30 | WT T6+/ pWBAD30 | WT T6+ cells with pWBAD30, pAB3 absent, Kan^R^ | This study |
| *Acinetobacter baumannii* ATCC 17978 T6+/ pWBAD30*mumT* | WT T6+/ pWBAD30*mumT* | WT T6+ cells with pWBAD30*mumT*, pAB3 absent, Kan^R^ | This study |
| *Acinetobacter baumannii* ATCC 17978 Δ*mumT* | Δ*mumT* | *mumT* k/o in *A. baumannii* ATCC 17978, pAB3 present | This study |
| *Acinetobacter baumannii* ATCC 17978 Δ*mumT*- pWBAD30 | Δ*mumT*- pWBAD30 | *mumT* k/o in *A. baumannii* ATCC 17978, pAB3 present, Kan^R^ | This study |
| *Acinetobacter baumannii* ATCC 17978 Δ*mumT*- pWBAD30mumT | Δ*mumT*- pWBAD30mumT | *mumT* k/o in *A. baumannii* ATCC 17978, pAB3 present, Kan^R^ | This study |
| *Acinetobacter baumannii* ATCC 17978 T6+ Δ*mumT* | Δ*mumT* T6+ | *mumT* k/o in *A. baumannii* ATCC 17978 T6+, pAB3 absent | This study |
| *Acinetobacter baumannii* ATCC 17978 Δ*absR28* | Δ*absR28* | *absR28* k/o in *A. baumannii* ATCC 17978, pAB3 present | This study |
| *Acinetobacter baumannii* ATCC 17978 Δ*absR28*-pWBAD30 | Δ*absR28*-pWBAD30 | *absR28* k/o in *A. baumannii* ATCC 17978 with pWBAD30, pAB3 present, Kan^R^ | This study |
| *Acinetobacter baumannii* ATCC 17978 Δ*absR28*-pWBAD30AbsR28 | Δ*absR28*-pWBAD30AbsR28 | *absR28* k/o in *A. baumannii* ATCC 17978 with pWBAD30AbsR28, pAB3 present, Kan^R^ | This study |
| *Acinetobacter baumannii* ATCC 17978 Δ*absR28*-pWBAD30AbsR28 mutant_M9 (U_27_C_28_C_29_U_30_) | Δ*absR28*- pWBAD30AbsR28 mutant_M9 (U_27_C_28_C_29_U_30_) | *absR28* k/o in *A. baumannii* ATCC 17978 with pWBAD30AbsR28 mutant_M9 (U_27_C_28_C_29_U_30_), pAB3 present, Kan^R^ | This study |
| *Acinetobacter baumannii* ATCC 17978 Δ*tssM* | Δ*tssM* | *tssM* k/o in *A. baumannii* ATCC 17978, pAB3 present | Lab stock |
| *Acinetobacter baumannii* ATCC 17978 T6+/pAB3 | WT T6+/pAB3 | T6SS repressed, pAB3 present, S/T^R^ | This study |
| *Acinetobacter baumannii* RPTC2 | RPTC2 | Clinical isolate | Dr. Varsha Gupta, GMCH, Chandigarh, India |
| *Acinetobacter baumannii* RPTC2 T6+ | RPTC2 T6+ | T6SS expressed | This study |
| *Acinetobacter baumannii* RPTC3 | RPTC3 | Clinical isolate | Dr. Varsha Gupta, GMCH, Chandigarh, India |
| *Acinetobacter baumannii* RPTC3 T6+ | RPTC3 T6+ | T6SS expressed | This study |
| *Escherichia coli* DH5α | *E. coli* DH5α | *supE44 hsdR17 recA1 endA1 gyrA96 thi-1*  *relA1* | Invitrogen, USA |
| *Escherichia coli* DH5α-pNYL GFP | *E. coli*-pNYL GFP | *E. coli*-pNYL GFP | Prof. N.K. Navani,  IIT Roorkee,  India |
| *Escherichia coli* J53 | *E. coli* J53 | *E. coli* J53 | Dr. Sanath Kumar H, ICAR-CIFE, India |
| *Pseudomonas aeruginosa* PAO1 | *P. aeruginosa* | *P. aeruginosa* | Prof. N.K. Navani,  IIT Roorkee,  India |

**Table S4.** Plasmids were used in this study.

| **Strains** | **Name used in this study** | **Description** | **Reference** |
| --- | --- | --- | --- |
| pUC18 | N/A | Cloning vector, Amp^R^ | Thermo Scientific,  USA |
| pMDIAI | N/A | Plasmid carrying apramycin (Apm) resistance cassette  flanked by FRT sites | Addgene |
| pUC18-UP*mumT*-AprFRT-DN*mumT* | N/A | *mumT* construct for *mumt* k/o cloned in pUC18, Amp^R^, Apm^R^ | This study |
| pUC18-UP*absR28*-AprFRT-DN*absR28* | N/A | *absR28* construct for *absR28* k/o cloned in pUC18, Amp^R^, Apm^R^ | This study |
| pUC18-UPAbaAL44-AprFRT-DN AbaAL44 | N/A | AbaAL44 construct for AbaAL44 k/o cloned in pUC18, Amp^R^, Apm^R^ | This study |
| pAT02 | N/A | Plasmid expressing *A. baumannii* RecT homolog,  Amp^R^ | Prof. Bryan Davies,  University of Texas,  San Antonio, USA |
| pAT03 | N/A | Plasmid expressing FLP recombinase enzyme  (flippase) for expression in *A. baumannii*, Amp^R^ | Prof. Bryan Davies,  University of Texas,  San Antonio, USA |
| pBAD30-Amp^R^ | N/A | Arabinose PBAD promoter, Amp^R^ | Prof. Eric D. Brown, McMaster University, Hamilton, ON |
| pBAD30-Kan^R^ | pBAD30 | Modified from pBAD30-Amp^R^, arabinose PBAD promoter, Kan^R^ | This study |
| pWBAD30-Kan^R^ | pWBAD30 | Modified from pBAD30-Kan^R^, arabinose PBAD promoter, A. baumannii compatible *ori* cloned, Kan^R^ | This study |
| pWBAD30-Kan^R^-*absR28* | pWBAD30-*absR28* | *absR28* cloned under PBAD promoter, Kan^R^ | This study |
| pWBAD30-Kan^R^-mumT | pWBAD30-mumT | *mumT* cloned under PBAD promoter, Kan^R^ | This study |
| pWBAD30-Kan^R^-*absR28* mutant-M9 (U_27_C_28_C_29_U_30_) | pWBAD30*absR28* mutant-M9 (U_27_C_28_C_29_U_30_) | *absR28* mutant-M9 cloned under PBAD promoter, Kan^R^ | This study |
| pUC18-M12 | N/A | *absR28* mutant (C_76_C_78_ to A_76_A_78_) cloned in pUC18 | This study |
| pUC18-M14 | N/A | *absR28* mutant (C_89_ to U_89_) cloned in pUC18 | This study |
| pUC18-M15 | N/A | *absR28* mutant (C_91_A_94_C_95_ to A_91_U_94_A_95_) cloned in pUC18 | This study |
| apUC18-M20 | N/A | *absR28* mutant (C_84_A_85_ to U_84_U_85_) cloned in pUC18 | This study |
| pUC18-*tssM** | N/A | Compensatory *tssM* mutant (C_84_A_85_ to U_84_U_85_) cloned in pUC18 | This study |

**Table S5.** Oligonucleotides were used in this study.

| **Primer name** | **Sequence (5**' **- 3**'**)** | **Description** | **Reference** |
| --- | --- | --- | --- |
| UP437bpFPmumT | ATGCGTCGACAATTAACTGAAGTGGC | Forward primer for cloning *mumT* 437 bp upstream into pUC18 | This study |
| UP437bpRPmumT | ACTCTAGATTAATGCGTTCCTCATCCATTTG | Reverse primer for cloning *mumT* 437 bp upstream into pUC18 | This study |
| DN500bpFPmumT | ATGGTACCAGACGACATGAATTGATAAG | Forward primer for cloning *mumT* 500 bp downstream into pUC18 | This study |
| DN500bpRPmumT | ATGGAATTCAACTCGTGCTTGCTCG | Reverse primer for cloning *mumT* 500 bp downstream into pUC18 | This study |
| UP125bpFPmumT | ATGGGGAACAGGGAATCTTTGTCAT | Forward primer for the amplification of *mumT* 125 bp upstream | This study |
| DN125bpRPmumT | ACCAGCATGAAAACCACAAGCAAT | Reverse primer for the amplification of *mumT* 125 bp downstream | This study |
| UP498bpFPAbsR28 | TAAGTCGACATATGCAACTACATTCATTGCTGC | Forward primer for cloning AbsR28 498 bp upstream into pUC18 | This study |
| UP498bpRPAbsR28 | TTATCTAGAGAACGGATTTTACCTGTTTT | Reverse primer for cloning AbsR28 498 bp upstream into pUC18 | This study |
| DN501bpFPAbsR28 | TATGGTACCAAATAAGAGAATAATTATGGGCAT | Forward primer for cloning AbsR28 501 bp downstream into pUC18 | This study |
| DN501bpRPAbsR28 | TATGAATTCCCTAAAGTGCCCAGCTGTTTT | Reverse primer for cloning AbsR28 501 bp downstream into pUC18 | This study |
| UP126bpFPAbsR28 | ATTAATTCCTTACGATCAAATGGATGTAAAACC | Forward primer for the amplification of AbsR28 126 bp upstream | This study |
| DN126bpRPAbsR28 | TGTGCCATTTTCTTGAGTTGTTCAATACTT | Reverse primer for the amplification of AbsR28 126 bp downstream | This study |
| AprF-Bam | ATCAGGATCCGTCGACCTGCAGTTC | Forward primer for cloning Apr-FRT into pUC18 | Lab stock |
| AprR-Kpn | ATGGTACCGTGTAGGCTGGAGCTGCTTC | Reverse primer for cloning Apr-FRT into pUC18 | Lab stock |
| UP500Abal44ko FP | GTAAGCTTGCTGCAT GTCCAGGAGTGAACATA | Forward primer for cloning Abal44 500 bp upstream into pUC18 | This study |
| UP500Abal44ko RP | ATGGATCCAAGAGAGGTTTTTATCATGCAATTA TC | Reverse primer for cloning Abal44 500 bp upstream into pUC18 | This study |
| DN500Abal44ko FP | ATGGTACCAAAGTATAAATAATTTAGAAACCAC | Forward primer for cloning Abal44 500 bp downstream into pUC18 | This study |
| DN500Abal44ko RP | TTGAATTCTTCATTTTGAAAATGACGTCCATTGAG | Reverse primer for cloning Abal44 500 bp downstream into pUC18 | This study |
| UP150Abal44ko FP | GCAGCTGGTTTTAAA TTGTATTTAACTGGT G | Forward primer for the amplification of Abal44 150 bp upstream | This study |
| DN125Abal44ko RP | GCGAACTGGTCAATT TATTTTGTCGG | Reverse primer for the amplification of Abal44 125 bp downstream | This study |
| Kan FP pWBAD30 | TTCGATCGGAACTTCAAGATCCCCTCAC | Forward primer for cloning Kanamycin resistance marker into pBAD30 | Lab stock |
| Kan RP pWBAD30 | TTCGATCGTTCTCGAGAAGTATAGGAACTTCAGAGC | Reverse primer for cloning Kanamycin resistance marker containing XhoI restriction site into pBAD30 | Lab stock |
| pW FP | TGTCTCGAGGATCGTAGAAATATCTATGATTATC | Forward primer for cloning *A. baumannii* ori into pBAD30-kan^R^ | Lab stock |
| pW RP | TGTCTCGAGGGATTTTAACATTTTGCGTTGTTC | Reverse primer for cloning *A. baumannii* ori into pBAD30-kan^R^ | Lab stock |
| *absR28* FP pWBAD30 | AGGAATTCGTCAAAAACTTGATCTTTAG | Forward primer for cloning *absR28* into pWBAD30-kan^R^ | This study |
| *absR28* RP pWBAD30 | CCCAAGCTTATTGTCCGAATAGGAATAAAAAAACCTAGCG | Reverse primer for cloning absR28 into pWBAD30-kan^R^ | This study |
| RT *mumT* FP | TGCCATGGATAAAAGCATGA | qRT-PCR forward primer for *mumT* | This study |
| RT *mumT* RP | CAACTGTACCGCCAACAATGG | qRT-PCR reverse primer for *mumT* | This study |
| RT *znuB* FP | ATTTGAGGCTGCCAATAGCG | qRT-PCR forward primer for *znuB* | This study |
| RT *znuB* RP | AAACGATGCTTTGCTTGCCC | qRT-PCR reverse primer for *znuB* | This study |
| RT *tonB* FP | CCAGATCCATCGCCAAAACG | qRT-PCR forward primer for *tonB* | This study |
| RT *tonB* RP | GGGTTACGCGCACGTTAGTA | qRT-PCR reverse primer for *tonB* | This study |
| RT *tssB* FP | TCAGCGAATTCGACCTCCAC | qRT-PCR forward primer for *tssB* | Lab stock |
| RT *tssB* RP | GTACGCTCAAGCTCAGATGC | qRT-PCR reverse primer for *tssB* | Lab stock |
| RT *tssC* FP | GTTGGTGTGCTGCTATTCGC | qRT-PCR forward primer for *tssC* | This study |
| RT *tssC* RP | CTCTTTTTCACGGCGATCCG | qRT-PCR reverse primer for *tssC* | This study |
| RT *hcp* FP | CTTCAAGTAGTGTAGGCGGC | qRT-PCR forward primer for *hcp* | Lab stock |
| RT *hcp* RP | CCATTTGCACGATAGAAGTC | qRT-PCR reverse primer for *hcp* | Lab stock |
| RT *tssE* FP | GTGGGGCTTTCTACAGCCAA | qRT-PCR forward primer for *tssE* | This study |
| RT *tssE* RP | ACCCGTATTTGTCTTAGCCGAG | qRT-PCR reverse primer for *tssE* | This study |
| RT *tssF* FP | TAGTAGCTTGGCGAGACGTG | qRT-PCR forward primer for *tssF* | Lab stock |
| RT *tssF* RP | GATCACACGCCACTGTTCAC | qRT-PCR reverse primer for *tssF* | Lab stock |
| RT *tssG* FP | ACCTGGTGCAGTCCAACTTT | qRT-PCR forward primer for *tssG* | This study |
| RT *tssG* RP | AAAAAGCGCCTTGCCCTAAG | qRT-PCR reverse primer for *tssG* | This study |
| RT *tssM* FP | CTCCGGCAACCAATCAGTCT | qRT-PCR forward primer for *tssM* | This study |
| RT *tssM* RP | AGCTGTAATACGAGCACCCG | qRT-PCR reverse primer for *tssM* | This study |
| RT *paar* FP | TGGCTAGCCCTTACATTACG | qRT-PCR forward primer for *paar* | Lab stock |
| RT *paar* RP | CGTTTTATGCGCCGGACAAG | qRT-PCR reverse primer for *paar* | Lab stock |
| RT *tssH* FP | CTCGAGTGCAATTATGCAGGC | qRT-PCR forward primer for *tssH* | This study |
| RT *tssH* RP | CACAACTCTCATGCGCCCTA | qRT-PCR reverse primer for *tssH* | This study |
| RT *tssA* FP | CAATCGCGAGCAAGCAATGA | qRT-PCR forward primer for *tssA* | This study |
| RT *tssA* RP | GCTAACCATTCATGCAGCGG | qRT-PCR reverse primer for *tssA* | This study |
| RT *tssK* FP | GCAGACCCACGAGTTGATTC | qRT-PCR forward primer for *tssK* | Lab stock |
| RT *tssK* RP | CTCACACCCGAACGTACTGG | qRT-PCR reverse primer for *tssK* | Lab stock |
| RT *tssL* FP | TAACCCAGCAAGACCCAAGC | qRT-PCR forward primer for *tssL* | This study |
| RT *tssL* RP | TCGCTCTTTTCCACGACTACG | qRT-PCR reverse primer for *tssL* | This study |
| RT *vgrG* FP | TGACCGTCCGTTTGTAGTGG | qRT-PCR forward primer for *vgrG* (A1S_0550) | This study |
| RT *vgrG* RP | TGACCGCATGGCTACTTTGT | qRT-PCR reverse primer for *vgrG* (A1S_0550) | This study |
| RT *tetR1* FP | ATGCTGTACTGCCTTTGTCTCT | Forward primer to check for *tetR1* in pAB3 | This study |
| RT *tetR1* RP | CCGTTTCGTGGTCCACACAT | Reverse primer to check for *tetR1* in pAB3 | This study |
| RT *tetR2* FP | CAACCTCTTGGGCCAGTGTG | Forward primer to check for *tetR2* in pAB3 | This study |
| RT *tetR2* RP | GGTCCACGTGCCACTGATAG | Reverse primer to check for *tetR2* in pAB3 | Lab stock |
| RT *absR1* FP | GGTTAAGTAAAGAATTTTAAAG | qRT-PCR forward primer for *absR1* | Lab stock |
| RT *absR1* RP | CTCTACCGAAGCAAAAGC | qRT-PCR reverse primer for *absR1* | Lab stock |
| RT *absR11* FP | AACGTAGCGGTGTCACATCA | qRT-PCR forward primer for *absR11* | Lab stock |
| RT *absR11* RP | GGTGAAGAGTCCCATTCCCT | qRT-PCR reverse primer for *absR11* | Lab stock |
| RT *absR25* FP | AAATCATGTGTAGGACCGAG | qRT-PCR forward primer for *absR25* | Lab stock |
| RT *absR25* RP | AAAGCCTACTCAAGAAGCAG | qRT-PCR reverse primer for *absR25* | Lab stock |
| RT *absR28* FP | AAGGAGGACATCATGCCAAC | qRT-PCR forward primer for *absR28* | Lab stock |
| RT *absR28* RP | AATTCAAGCATTCGGACAAG | qRT-PCR reverse primer for *absR28* | Lab stock |
| RT *absR29* FP | CGCAGTCAATCAATCAGTGCATTT | qRT-PCR forward primer for *absR29* | Lab stock |
| RT *absR29* RP | GATGCAAAGAGCTTGCCAAT | qRT-PCR reverse primer for *absR29* | Lab stock |
| RT *hfq* FP | CCTTGACTACCACCCTGAGC | qRT-PCR forward primer for *hfq* | Lab stock |
| RT *hfq* RP | TCTACAGTTGTTCCAGCTCGT | qRT-PCR reverse primer for *hfq* | Lab stock |
| RT 16s FP | AGAGGGTGCGAGCGTTAATC | qRT-PCR housekeeping gene forward primer | Lab stock |
| RT 16s RP | GTTAAGCTCGGGGATTTCAC | qRT-PCR housekeeping gene reverse primer | Lab stock |
| RT A1S_2838 FP | GGCACCATTCGTAGGTGGTT | Forward primer to check the polar effect of Δ*absR28* upstream | This study |
| RT A1S_2838 RP | AGCGGAATCGTCTTCTTCGG | Reverse primer to check the polar effect of Δ*absR28* upstream | This study |
| RT A1S_2839 FP | ATCCGGGTCTTGTCCGAATG | Forward primer to check the polar effect of Δ*absR28* downstream | This study |
| RT A1S_2839 RP | CCTGAATGGAGCATCACCCA | Reverse primer to check the polar effect of Δ*absR28* downstream | This study |
| *absR28* IVT FP | CCGGAATTCTAATACGACTCACTATAGGGAGATTTTCAACGGCAC | Forward primer for *absR28* *in vitro* transcription | This study |
| *absR28* IVT RP | CCCAAGCTTATTGTCCGAATAGGAATAAAAAAACCTAGCG | Reverse primer for *absR28* *in vitro* transcription | This study |
| *absR28*-M9 IVT FP | CCGGAATTCTAATACGACTCACTATAGGGAGATTTTCAACGGCACTTTTTAAAAA**TCCT**GGACATC | Forward primer for *absR28*-M9 *in vitro* transcription | This study |
| *tssM* IVT FP | TAATACGACTCACTATAGGGGTTTGCACAAACATCTGTTGAACCA | Forward primer for *tssM* *in vitro* transcription | This study |
| *tssM* IVT RP | CGTACTCTGCTTGGGTATCCTTTT | Reverse primer for *tssM* *in vitro* transcription | This study |
| *pntB* IVT FP | CCGGAATTCTAATACGACTCACTATAGGGGGCCTGCCTGTACTTGG | Forward primer for *pntB* *in vitro* transcription | This study |
| *pntB* IVT RP | CCCAAGCTTCACCACCAATCATCCAAATAACCGG | Reverse primer for *pntB* *in vitro* transcription | This study |
| MumT-pWBAD-FP | TTGGGCTAGCGAATTCGCTATTTCTTGAATGCTTTTTATCC | Forward primer for *mumT* complementation | This study |
| MumT-pWBAD-RP | TACCGAGCTCGAATTCTTAAAGCTTGGTCAAATAATTAAAT | Reverse primer for *mumT* complementation | This study |
| *absR28*-M12 FP | CGATTTGGAAAGATGTCAGCTCACGGACAAG | Forward primer for *absR28* mutation (A_76_A_78_) | This study |
| *absR28*-M12 RP | GCTGACATCTTTCCAAATCGTTACTGTGTTTTAC | Reverse primer for *absR28* mutation (A_76_A_78_) | This study |
| *absR28*-M14 FP | GTCAGCTTACGGACAAGTGTACAAATTTAATTCACTTG | Forward primer for *absR28* mutation (U_89_) | This study |
| *absR28*-M14 RP | CACTTGTCCGTAAGCTGACATCGTGCCAAATC | Reverse primer for *absR28* mutation (U_89_) | This study |
| *absR28*-M15 FP | CAGCTCAAGGTAAAGTGTACAAATTTAATTCAC | Forward primer for *absR28* mutation (A_91_U_94_A_95_) | This study |
| *absR28*-M15 RP | GTACACTTTACCTTGAGCTGACATCGTGCC | Reverse primer for *absR28* mutation (A_91_U_94_A_95_) | This study |
| *absR28*-M20 FP | CACGATGTTTGCAAACGGACAAGTGTACAAATTTAA | Forward primer for *absR28* mutation (U_84_U85) | This study |
| *absR28*-M20 RP | CTTGTCCGTTTGCAAACATCGTGCCAAATCGTTACTG | Reverse primer for *absR28* mutation (U_84_U85) | This study |
| *tssM* compensatory F1-FP | ACGACGGCCAGTGCCAAGCTTGTTTGCACAAACATCTGTTGAACC | Forward primer for fragment 1 of *tssM* compensatory mutation | This study |
| *tssM* compensatory F1-RP | AGACATCAATGCCACGAACAATGACAAGGC | Reverse primer for fragment 1 of *tssM* compensatory mutation | This study |
| *tssM* compensatory F2-FP | TGTTCGTGGCATTGATGTCTAGGAATACTTCAATT | Forward primer for fragment 2 of *tssM* compensatory mutation | This study |
| *tssM* compensatory F2-RP | TATGACCATGATTACGAATTCCGTACTCTGCTTGGGTATCCT | Reverse primer for fragment 2 of *tssM* compensatory mutation | This study |
